# Self‐Propelled Enzymatic Nanomotors from Prodrug‐Skeletal Zeolitic Imidazolate Frameworks for Boosting Multimodel Cancer Therapy Efficiency

**DOI:** 10.1002/advs.202301919

**Published:** 2023-05-15

**Authors:** Jieyu Yu, Yan Li, An Yan, Yuwei Gao, Fei Xiao, Zhengwei Xu, Jiayun Xu, Shuangjiang Yu, Junqiu Liu, Hongcheng Sun

**Affiliations:** ^1^ College of Material Chemistry and Chemical Engineering Key Laboratory of Organosilicon Chemistry and Material Technology Ministry of Education Key Laboratory of Organosilicon Material Technology of Zhejiang Province Hangzhou Normal University Hangzhou Zhejiang 311121 P. R. China

**Keywords:** cascade enzyme reactions, cisplatin skeleton, enzymatic nanomotors, O_2_ self‐supply, synergetic cancer therapy

## Abstract

Self‐propelled nanomotors, which can autonomous propelled by harnessing others type of energy, have shown tremendous potential as drug delivery systems for cancer therapy. However, it remains challenging for nanomotors in tumor theranostics because of their structural complexity and deficient therapeutic model. Herein, glucose‐fueled enzymatic nanomotors (GC6@cPt ZIFs) are developed through encapsulation of glucose oxidase (GOx), catalase (CAT), and chlorin e6 (Ce6) using cisplatin‐skeletal zeolitic imidazolate frameworks (cPt ZIFs) for synergetic photochemotherapy. The GC6@cPt ZIFs nanomotors can produce O_2_ through enzymatic cascade reactions for propelling the self‐propulsion. Trans‐well chamber and multicellular tumor spheroids experiments demonstrate the deep penetration and high accumulation of GC6@cPt nanomotors. Importantly, the glucose‐fueled nanomotor can release the chemotherapeutic cPt and generate reactive oxygen species under laser irradiation, and simultaneously consume intratumoral over‐expressed glutathione. Mechanistically, such processes can inhibit cancer cell energy and destroy intratumoral redox balance to synergistically damage DNA and induce tumor cell apoptosis. Collectively, this work demonstrates that the self‐propelled prodrug‐skeleton nanomotors with oxidative stress activation can highlight a robust therapeutic capability of oxidants amplification and glutathione depletion to boost the synergetic cancer therapy efficiency.

## Graphical abstract

A dual‐enzyme‐engineered therapeutic GC6@cPt ZIF nanomotors are developed through encapsulation of glucose oxidase, catalase, and chlorin e6 using cisplatin‐skeletal zeolitic imidazolate frameworks (cPt ZIFs). Such nanomotors can not only facilitate the deep penetration in tumor tissue, but also enhance the combined chemotherapy and photodynamic therapy through boosting oxidative stress and glutathione depletion.

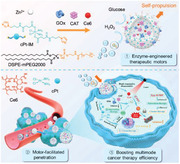

## 1 Introduction

Motors are devices that can generate autonomous propulsion by converting others type of sources into mechanical motion,^[^ ^1^, ^2^ ^]^ which have showed great promise for addressing many challenges in the field of sensing, environmental remediation, and even biomedicine.^[^ ^3^, ^4^ ^]^ Especially for cancer therapy, recent advances in micro/nanomotor research have made great progress in minimally invasive surgery, active drug delivery, and others due to their autonomous motion behavior and tunable functionality.^[^ ^5^ ^]^ As an upgrade version of drug delivery platform, a variety of micro/nanomotors, driven by light,^[^ ^6^ ^]^ heat,^[^ ^7^ ^]^ magnetic fields,^[^ ^8^ ^]^ ultrasounds,^[^ ^9^ ^]^ chemical reactions,^[^ ^10^, ^11^ ^]^ or by living organisms,^[^ ^12^, ^13^ ^]^ have been well developed for their self‐propulsion and delivering drug on‐command, and have demonstrated great superiority for cancer therapy. Among them, chemical motors that converting localized chemical energy into mechanical energy using self‐destructive reactions or catalyzed reactions is the most developed item for cancer therapy due to their microenvironmental response. Although it can bring revolutionary progress to traditional therapeutic model, the chemically driven nanomotor platform for in vivo cancer therapy is still in the embryonic stage and far from clinical application for their structural complexity and deficient therapeutic model.^[^ ^14^ ^]^

Enzymes are naturally existed biological catalysts that convert substrates to products. By means of enzyme‐triggered biocatalytic reactions, enzymatic micro/nanomotors, which anchoring enzymes on micro/nanosized scaffolds, can enzymatically generate active motion for self‐propulsion under mild conditions.^[^ ^15^, ^16^ ^]^ Due to the good biocompatibility and biologically available fuels, such versatile micro/nanomotors have aroused widespread concerns in recent years. Meanwhile, enzymatic micro/nanomotors can produce toxic bioproducts, such as hydrogen peroxide,^[^ ^17^ ^]^ and can be further used for synergistic biomedical applications. Up to now, numerous kinds of enzymes, such as catalase (CAT),^[^ ^18^ ^]^ glucose oxidase (GOx),^[^ ^19^ ^]^ urease,^[^ ^20^ ^]^ or their combination,^[^ ^21^ ^]^ have been successfully employed for developing enzyme‐engineered micro/nanomotors. Such motors rely on the enzymatic catalysis of biological available substrates as fuels to generate gas bubbles for propelling directed motion rather random Brownian motion.

As we know, the regulation of intracellular oxidative stress is one of the main strategies for cancer therapy, because accumulation of reactive oxidative species (ROS), including superoxide (O_2_^•−^), hydrogen dioxide (H_2_O_2_), singlet oxygen (^1^O_2_), and hydroxyl radicals (•OH), or depletion of glutathione (GSH) can cause the cell apoptosis through mitochondrial dysfunction or ferroptosis activation.^[^ ^22^, ^23^ ^]^ In recent years, photodynamic therapy (PDT) has been emerged as a fashionable approach for cancer therapy for their high therapeutic effect and minimum invasiveness.^[^ ^24^, ^25^ ^]^ It involves the integration of photosensitizer, light source, and localized oxygen (O_2_), and can produce highly reactive ^1^O_2_ from O_2_ under laser irradiation for direct tumor cell ablation, vascular damage, and stimulation of antitumor immune response.^[^ ^26^ ^]^ However, the PDT efficacy in solid tumor tissues is greatly restricted by their hypoxia characteristic (less than 5 mm Hg) and overproduced intracellular antioxidant glutathione (GSH, up to 10 × 10^−3^ m).^[^ ^27^ ^]^ In order to enabling the PDT efficacy, many researches have been devoted for the ^1^O_2_ augmentation through direct carrying or in situ generation of O_2_ in tumor tissues, or the GSH depletion to block their consumption.^[^ ^28^, ^29^, ^30^ ^]^

Intriguingly, CAT‐engineered micro/nanomotors can consume overproduced endogenous hydrogen peroxide (H_2_O_2_, about 100 × 10^−6^ to 1 × 10^−3^ m) in tumor cells to generate oxygen (O_2_) for their self‐propulsion. Meanwhile, the supplied O_2_ species can further enable the O_2_‐dependent PDT therapy through reversing the tumor hypoxia surroundings. What is more, the regulation of the redox stress can also reverse the multiple drug resistance of tumor cells with improved chemotherapy.^[^ ^31^, ^32^ ^]^ Thus, an expansion of de novo self‐propelled enzymatic motors with regulated oxidative stress will enable a better understanding of the mechanisms of motors for in vivo synergetic cancer therapy.

**Scheme 1:**
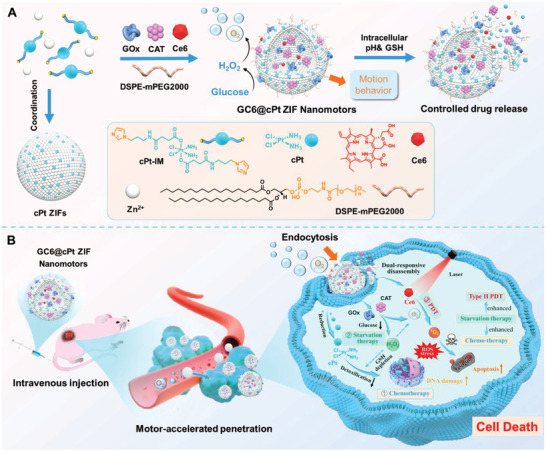
Tailoring enzyme‐engineered cisplatin prodrug nanomotors for synergistic cancer therapy of breast cancers. A) Cisplatin‐backboned ZIF nanomotors for encapsulation and dual‐responsive release of GOx, CAT, and Ce6. B) Schematic illustration of the GC6@cPt ZIF motors for synergistic cancer therapy with combined starvation, photodynamic, and chemo therapy.

Herein, we tailed a dual‐responsive prodrug‐skeletal enzymatic nanomotor strategy based on zeolitic imidazolate frameworks (ZIFs) for ROS‐potentiated synergetic cancer therapy with combined chemo‐, starvation, and dynamic therapy. Cisplatin (cPt) is a widely used therapeutic agent through intra‐ and interstrand cross‐links on DNA, while pseudo‐octahedral cPt prodrugs Pt(IV) by chemical oxidation exhibit less potency and toxicity for their enhanced inertia to ligand substitution.^[^ ^33^ ^]^ Such cPt prodrug can be reduced to cPt Pt(II) with enhanced cytotoxicity within the reductive tumor surroundings. As shown in **Scheme** **1**A, a cisplatin prodrug‐skeletal ZIF (cPt ZIF) was first developed through metal coordination assembly between imidazole‐terminated cPt prodrug (cPt‐IM) and zinc ion (Zn^2+^).^[^ ^34^ ^]^ The dual GOx and CAT enzymes and photosensitizer chlorin e6 (Ce6) were anchored on the ZIF structures through in situ encapsulation strategy to develop enzyme‐engineered GC6@cPt ZIF nanomotors. The GC6@cPt ZIF nanomotors can generate O_2_ from endogenous glucose and H_2_O_2_ by enzymatic cascade reactions to rapidly propel the self‐propulsion of nanomotors to targeted tumor tissues with improved penetration depth. When arrived at tumor cells, the cPt ZIF skeletons were nanomotors collapsed with the chemotherapeutic cPt drug and cargoes being released in response to the overexpressed intratumoral acid species (pH 6.5–6.9) and reductive GSH (Scheme 1B).^[^ ^35^ ^]^ In this stage, overproduced intratumoral H_2_O_2_ was decomposed by CAT to generate O_2_, which was developed for O_2_‐dependent PDT therapy.^[^ ^36^ ^]^ Meanwhile, intracellular H_2_O_2_ can be continuously supplied by GOx‐catalyzed consumption of glucose with gluconic acid as by‐product. The acid accumulation and the consumption of the glucose can be further employed for accelerated drug release and starvation therapy. Meanwhile, the upregulated oxidative stress with synchronously depleted GSH can further evoke the PDT efficacy and the DNA damage chemotherapeutic cPt drug.^[^ ^31^, ^37^, ^38^ ^]^ Through combining the concepts of oxidative stress regulation, self‐propelled enzymatic nanomotors, and synergistic cancer therapy, the implementation of GC6@cPt ZIF nanomotors will provide theoretical guidance and technical basis for intelligent nanomotors for in vivo cancer therapy.

## 2 Results and Discussion

### 2.1 Preparation and Characterization of GC6@cPt ZIF Nanomotors

**Figure 1:**
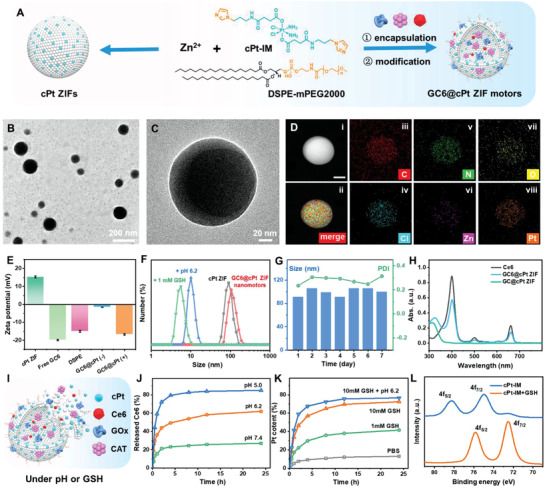
Preparation and characterization of GC6@cPt ZIF nanomotors. A) Schematic illustration for the preparation of cPt ZIFs and GC6@cPt ZIF nanomotors. B) TEM image and C) magnified HR‐TEM image of GC6@cPt ZIF nanomotors. D) i,ii) Dark‐field HR‐TEM and corresponding EDX elemental mappings of GC6@cPt ZIF nanomotors; iii) C, iv) N, v) O, vi) Cl, vii) Zn, and viii) Pt, the scale bar is 50 nm. E) Zeta‐potentials of cPt ZIF, free GC6, DSPE‐PEG2000, GC6@cPt ZIF nanomotors without (−) and with (+) DSPE‐PEG2000. F) Size distribution of cPt ZIFs (gray), GC6@cPt ZIFs (red), and GC6@cPt ZIFs (red) after treatment with GSH (green) or acid (6.2). G) Size change and PDI of GC6@cPt ZIF nanomotors after 7 days measurements. H) Absorption spectra of Ce6 (gray), cPt ZIFs (green), and GC6@cPt ZIF nanomotors (cyan). I) Schematic representation of the GC6@cPt ZIFs under acidic condition and reductive GSH medium for controlled release of cPt, Ce6, GOx, and CAT. J) Ce6 release profiles of GC6@cPt ZIF nanomotors in PBS under pH 7.4, 6.2, and 5.0 for 24 h. K) Pt element release profiles of GC6@cPt ZIF nanomotors in PBS (pH 7.4) with different amount of GSH (0 × 10^−3^, 1.0 × 10^−3^, 10 × 10^−3^ m) and in PBS (pH 6.2) with 10 × 10^−3^ m GSH. L) XPS curves of Pt_4f_ before and after incubation with GSH (5 × 10^−3^ m) for 4 h.

In order to prepare dual‐responsive ZIF nanomotors with cPt‐based skeletons, an imidazole‐terminated cisplatin prodrug (cPt‐IM) was first synthesized by PyBOP‐catalyzed amidation reaction of dicarboxyl cisplatin prodrug (cPt‐COOH) and (3‐aminopropyl)imidazole, as illustrated in Scheme S1 in the Supporting Information, and its molecular structure was also confirmed by ^1^H NMR spectra and liquid chromatograph‐mass spectrometry (LC‐MS, Figures S1–S3, Supporting Information).^[^ ^39^ ^]^ As schematically illustrated in **Figure** **1**A (left), a water‐soluble cPt ZIF nanoparticle was constructed using a bottom‐up approach with the coordination of cPt‐IM ligands and Zn^2+^ in the presence of an amphiphilic block copolymer 1,2‐distearoyl‐*sn*‐glycero‐3‐phosphoethanolamine‐*N*‐[methoxy(polyethylene glycol)‐2000] (DSPE‐mPEG2000). It was shown from the transmission electron microscopy (TEM) and the atomic force microscopy measurements that cPt ZIF nanoparticles afforded hollow spherical shapes were formed (Figure S4, Supporting Information), which might be the reason that the initially coordinated Zn/cPt‐IM clusters was disassembled and rearranged at the surface into a well‐organized cPt ZIFs shells.^[^ ^40^ ^]^ Such hollow structures can facilitate the high‐loading encapsulation of different cargoes for advanced applications.

In order to encapsulating enzymes and molecular cargoes, the same procedure is happened in the presence of GOx, CAT, and photosensitizer Ce6 for the preparation of GC6@cPt ZIF nanomotors (Figure 1A right). It was found form high‐resolution (HR)‐TEM results (Figure 1B,C) that the GC6@cPt ZIF nanomotors exhibited the quite similar morphologies with the cPt ZIF nanoparticles, and the monodispersed size about 70 to 120 nm. Further, energy‐dispersive X‐ray spectroscopy (EDX) elemental mappings of GC6@cPt ZIF nanomotors reveal the uniformly distributed C, N, O, Cl, Zn, and Pt elements throughout the entire architectures (Figure 1D). Besides, the zeta potential was further measured to evaluate each procedure during the formation of frameworks. As shown in Figure 1E, the surface potential of cPt ZIF was 15.3 mV owing to the positive charge of coordinative structures. Because of the negatively charged properties of GOx/CAT/Ce6 cargoes (free GC6, −19.9 mV), the GC6@cPt ZIF nanomotors showed a nearly neutralized zeta potential with value of about −1.50 mV, which was also validated the efficient construction of GC6@cPt ZIFs. After employing DSPE‐PEG2000 (−15.0 mV) when making the GC6@cPt ZIF nanomotors, it exhibited significantly negatively charges (−16.7 mV) than that of GC6@cPt ZIFs without DSPE‐PEG2000. Such negatively charged potential helps the GC6@cPt ZIF nanomotors to well dispersion in aqueous medium.

Besides, as shown in dynamic light scattering measurement in Figure 1F, the existence of numerous cargoes did not obviously affect the formation of ZIF frameworks. The GC6@cPt ZIF nanomotors also showed the comparable structures and dimensions with the cPt ZIFs. The hydrodynamic size was measured to be 122 nm, which was a little bit larger than the TEM results because of the hydrodynamic layer. Moreover, the particle size and the polydispersity index (PDI) of GC6@cPt ZIF nanomotors remained steady during the 7 days observation even in phosphate‐buffered saline (PBS, pH = 7.4, Figure 1G), which indicated its high stability under physiological condition. After that, the contents of photosensitizer Ce6 and Pt element in GC6@cPt ZIF nanomotors were identified by the UV‐vis and the inductively coupled plasma‐optical emission spectrometry (ICP‐OES) measurements, respectively. The characteristic absorption peak of Ce6 at 642 nm could be simultaneously observed in the UV‐vis spectrum of GC6@cPt ZIF nanomotors (Figure 1H). According to the standard curves of Ce6 absorption (Figure S5A, Supporting Information) and the Pt elements (Figure S5B, Supporting Information), the Ce6 content and the Pt content were determined to be about 1.3 ± 0.3% and 8.42%. In addition, the final concentration of encapsulated GOx and CAT was determined to be 5.1% according to the standard curve with bicinchoninic acid (BCA) protein assay kit (Figure S6, Supporting Information).

Besides, tumor surroundings afford hyperacid and overexpressed reductive agents, the dual reduction‐ and acid‐sensitive release of cisplatin and agents is a critical step in the current design (Figure 1I). As we know, the acidic tumor microenvironment (TME) can breaks ZIF frameworks and can makes cargoes being released. It was found that the GC6@cPt ZIF nanomotor showed pH‐dependent cargo release profile and more than 80% cargo was released under the simulated tumor surrounding (pH 5.0) after 24 h, while only ≈20% for normal cells (pH = 7.4) (Figure 1J). In addition, the GSH/pH dual responsive release of cisplatin from this GC6@cPt ZIF nanomotor was investigated by the ICP‐OES. It was found that the cumulative Pt contents released from GC6@cPt ZIF scaffold were nearly 72% and 77% when incubated with 10 × 10^−3^ m GSH under pH 7.4 and 6.2, responsively, while that was only 13% in PBS buffer (pH = 7.4). To monitor the released Pt product, X‐ray photoelectron spectroscopy (XPS), ^195^Pt NMR, and LC‐MS measurements were further employed. Intriguingly, the Pt(IV) in cPt‐IM prodrug (binding energies for Pt_4f_, 78.2 and 75.0 eV) was completely reduced to Pt(II) (binding energies for Pt_4f_, 75.9 and 72.5 eV) from XPS results (Figure 1L). Meanwhile, according to the ^195^Pt NMR and LC‐MS results (Figures S7 and S8, Supporting Information), the ^195^Pt chemical shift of cPt‐IM prodrugs was shifted from 1230 pm to the location of −2340 ppm, the same value to the cisplatin, in the presence of 5 × 10^−3^ m reduced GSH.^[^ ^41^ ^]^ The molecule‐to‐charge ratio of the final product was measured to be 300.86 ([M+H]^+^) after GSH reduction, which indicating that the cisplatin drug was released from cPt‐IM prodrugs.^[^ ^42^ ^]^ Besides, the encapsulation of enzymes in cPt ZIF scaffolds was helpful for well maintaining their enzymatic activities (Figure S9, Supporting Information).

### 2.2 Propulsion Sources of GC6@cPt ZIF Nanomotors

**Figure 2:**
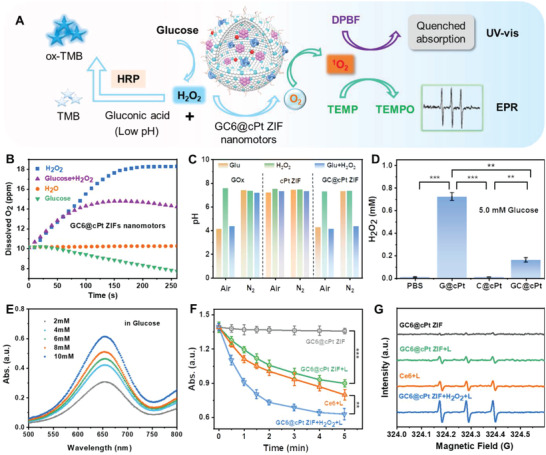
Properties and controlled release of GC6@cPt ZIF nanomotors. A) Schematic representation of the GC6@cPt ZIF nanomotors that cascade catalyze glucose into final product O_2_. H_2_O_2_, H^+^, and produced O_2_ were employed to evaluate the reaction progress. Change of B) dissolved O_2_, C) solution pH, and D) H_2_O_2_ level of reaction medium treated with different groups. E) Optical absorption spectra of HRP/TMB assay in glucose solution for 30 min. Before that, glucose solution with different concentrations (2 × 10^−3^, 4 × 10^−3^, 6 × 10^−3^, 8 × 10^−3^, and 10 × 10^−3^ m) was incubated with G@cPt ZIFs for 30 min. F) The absorbance of DPBF at different time points and G) EPR signal intensity of TEMP of Ce6+L, GC6@cPt ZIF, GC6@cPt ZIF+L, and GC6@cPt ZIF+H_2_O_2_+L groups to reflect the ^1^O_2_ generation efficiency.

Because of the porous frameworks of GC6@cPt ZIF nanomotors, the substrates can be allowed to arrive at the enzyme sites for enzymatic reaction. The encapsulated GOx in GC6@cPt ZIFs can be used to decompose the surrounding glucose into gluconic acid and hydrogen peroxide (H_2_O_2_), and can be further consumed by CAT to generate O_2_ through dual enzyme cascade reactions, as illustrated in **Figure** **2**A. The cascade reaction of nanomotors further promote the remodel of intracellular microenvironment and O_2_‐sufficient PDT.^[^ ^43^ ^]^ The whole processes of starvation and enhanced PDT upon light irradiation can be instructed as the following equations

(1) $$\mathrm{Glucose}+O_{2}\overset{\mathrm{GOx}}{\to }\mathrm{Gluconic}\;\mathrm{acid}+H_{2}O_{2}$$

(2) $$H_{2}O_{2}\overset{\mathrm{CAT}}{\to }H_{2}O+O_{2}$$

(3) $$O_{2}\overset{\mathrm{Ce}6,\;660\;\mathrm{nm}}{\to }{}^{1}O_{2}$$

The differentiation of pH value, dissolved O_2_ level, and generated H_2_O_2_ content were tested to evaluate the enzymatic reactions of GC6@cPt ZIF nanomotors. The G@cPt ZIFs and the C@cPt ZIFs with only GOx and CAT encapsulation, respectively, were developed for comparison using the similar procedure. It was evidenced from the dissolved O_2_ level that although the dissolved O_2_ was downregulated when incubated GC6@cPt ZIF nanomotors in pure glucose solution for the O_2_‐consumed production of H_2_O_2_ and gluconic acid, while it is gradually upregulated in the binary glucose/H_2_O_2_ surrounding (Figure 2B and Figure S10, Supporting Information), which can well simulate the real TME. In addition, such standpoint can be also evidenced from the decrease of pH value in Figure 2C and Figure S11 in the Supporting Information. Both glucose and pre‐existing O_2_ are indispensable for the dual enzymatic cascade reactions. Intriguingly, the downregulation of pH value will further facilitate the collapse of GC6@cPt ZIF nanomotors to release cargoes for exerting their therapeutic efficacies.

Besides, the defined concentration of produced H_2_O_2_ can be quantitatively identified by the horseradish peroxidase (HRP)‐catalyzed tetramethylbenzidine (TMB) oxidation assay with the regression equation of standard H_2_O_2_ curve (Figure S12, Supporting Information). As shown in Figure 2D, the G@cPt ZIFs could generate almost 0.78 × 10^−3^ m of H_2_O_2_ under 5 × 10^−3^ m glucose surroundings, while it was hardly detected in PBS group and C@cPt ZIFs. For the GC6@cPt ZIF nanomotors, it was only detected to be about 0.15 × 10^−3^ m under the same condition because of the CAT consumption. It was also found that the oxidation degree of TMB was gradually restricted when incubated C@cPt ZIF (100 µg mL^−1^) into 1 × 10^−3^ m H_2_O_2_ medium with extended incubation time (from 1 to 10 min), which was due to the competitive H_2_O_2_ consumption of C@cPt ZIF with HRP/TMB chromogenic reaction (Figure S13A, Supporting Information). Also, the blue color of TMB solution in optical image was rapidly turned to colorless in the presence of C@cPt ZIFs (Figure S13B, Supporting Information). In addition, the prolonged incubation time of G@cPt ZIF (100 µg mL^−1^) in glucose (5 × 10^−3^ m) and elevated glucose concentration (from 2 × 10^−3^ to 10 × 10^−3^ m) could accelerate the HRP‐catalyzed TMB oxidation from the UV‐vis absorption and the dynamic curve at 652 nm, because of the continuous production of H_2_O_2_ species (Figure 2E and Figure S14, Supporting Information).

Because of the co‐encapsulation of Ce6, the CAT‐catalyzed generation of O_2_ in GC6@cPt ZIF nanomotor could be employed to facilitate the PDT progress. The ^1^O_2_ production of GC6@cPt ZIF motors under 660 nm laser irradiation was first detected by diphenylisobenzofuran (DPBF) reagents. As shown in Figure 2F and Figure S15 in the Supporting Information, it is found that the ^1^O_2_ production of GC6@cPt ZIF nanomotor in 1 × 10^−3^ m H_2_O_2_ is much higher than that of GC6@cPt ZIF nanomotor without 1.0 × 10^−3^ m H_2_O_2_ under laser irradiation, because of the continuous supply of O_2_ species. However, the GC6@cPt ZIF nanomotor has a much lower ^1^O_2_ production if without laser irradiation. Such results indicated the continuous supply of O_2_ might facilitate the PDT efficacy. Besides, electron spin resonance spectroscopy also showed strong triplet peaks at ratio of 1:1:1 for laser‐triggered GC6@cPt ZIF nanomotors in H_2_O_2_ solution when 2,2,6,6‐tetramethylpiperidine (TEMP) was used as indicator, which gave us direct evidence to confirm the efficient production of ^1^O_2_ (Figure 2G).

### 2.3 Motion Behavior of GC6@cPt ZIF Nanomotors

**Figure 3:**
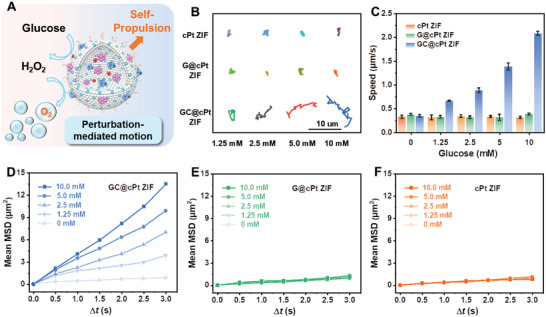
Analysis of the motion capability of the enzyme‐propelled GC6@cPt ZIF nanomotors. A) Schematic representation of the GC@cPt ZIF nanomotors that catalyze glucose into O_2_ resulting in self‐propulsion. B) Representative tracking trajectories of cPt ZIFs, G@cPt ZIF nanomotors, and GC@cPt ZIF nanomotors with different glucose concentrations. C) Average speed of different groups obtained by optical tracking in different glucose medium (*N* = 15). Averaged MSDs of the different types of D) GC@cPt ZIFs, E) G@cPt ZIFs, and F) cPt ZIFs in different glucose medium.

It has proved that the generation of gaseous bubbles around nanoparticles could cause force imbalance around the nanoparticles, which could facilitate the “self‐propulsion” rather than the *Brownian* movement (**Figure** **3**A).^[^ ^44^ ^]^ Optical microscopy was carried out to analyze the motion behavior of the GC6@cPt nanomotor and their control group (cPt ZIF and G@cPt ZIF). As shown in Figure 3B, the presence of glucose can be fueled by dual enzyme cascades in GC6@cPt ZIF nanomotor to generate gluconic acid and O_2_ to achieve self‐propulsion. Unlike the previously reported Janus nanomotors with predicted motion direction and rapid motion behavior, such one‐step synthetic nanomotors can be also propelled using the differential disturbing force around the nanomotors.^[^ ^45^ ^]^ To study the autonomous movement of GC6@cPt ZIF nanomotors, nanomotors’ trajectory was tracked to record and analyze the real‐time movement. GC6@cPt ZIF nanomotors without fuel exhibited the typical *Brownian* motion with randomly controlled immigration, while behavior as “self‐propelled nanomotors” with enhanced unidirectional movement with the presence of glucose.

As shown in Figure 3C, the average speed of GC6@cPt ZIF nanomotor also indicated a linear relationship with the glucose concentration. The motion speed can reach to a maximum 2.08 µm s^−1^ for GC6@cPt motor at 10 × 10^−3^ m glucose, while that was only 0.31 and 0.38 µm s^−1^ for nonmotor cPt ZIF and G@cPt ZIF groups, respectively. Based on the trajectory records in Figure 3B, the mean squared displacement (MSD) plots with different concentrations of glucose were extracted and showed always increased linearly with the time interval (Δ*t*) (Figure 3D). For comparison, the corresponding MSD plots of cPt ZIFs and G@cPt ZIFs were showed nonrelevant to the glucose concentration (Figure 3E,F). All these results indicated that the GC6@cPt motors can be propelled by glucose, which might be advantageous in penetration and internalization for cancer therapy.

### 2.4 Deep Penetration and Accumulation in Tumors

**Figure 4:**
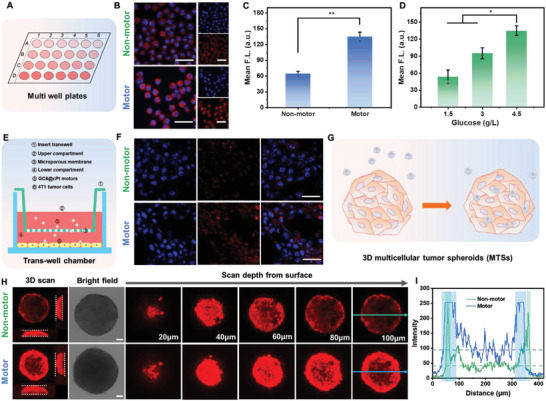
In vitro penetration test for GC@cPt ZIF motors. A) Schematic diagram of the multi‐well plate experiment for penetration ability test. B) CLSM fluorescence images and C) quantitative fluorescence analysis of 4T1 cells when incubated with Rh B‐labeled nonmotor (cPt ZIF, up) and motor (GC@cPt ZIF motor, down). Cell nucleus was stained by 4′,6‐diamidino‐2‐phenylindole (blue). D) Quantitative fluorescence analysis of 4T1 cells when incubated with motor group (GC@cPt ZIF motor) in DMEM medium with 1.5, 3.0, and 4.5 g L^−1^ of glucose concentration. E) Schematic representative of the trans‐well experiment for penetration ability test. F) CLSM fluorescence images of nonmotor (up) and motor (down) through the trans‐well experiment model. G) Schematic representative of the 3D MTSs experiment for penetration ability test. H) CLSM images of 3D MTSs incubated with cPt ZIF nonmotor group (up) and GC@cPt ZIF motor group (down) for 8 h and CLSM images for in‐depth scanning. I) Quantitative fluorescence analysis of CLSM images on the 100 µm depth by using the plot profile of image J. The scale bar is 50 µm.

With the self‐propelled feature, the GC6@cPt ZIF nanomotors might be facilitated to penetrate efficiently into the tumor tissues with enhanced infiltrating depth and accumulation. We evaluated the cell uptake behavior of the GC@cPt ZIF nanomotor on multi‐well plate through labeling with Rhodamine B isothiocyanate (RITC), and cPt ZIF was selected as nonmotor group for comparison (**Figure** **4**A). Due to the self‐propelled penetration, the GC@cPt ZIF nanomotor group showed brilliant red fluorescence in cytoplasm, while cPt ZIF nonmotor group only displayed faint fluorescence under the same condition (Figure 4B). Statistically analyzing the average fluorescence intensity, the motor group showed twice higher intensity than nonmotor groups, demonstrating the motor facilitated penetration into tumor cells (Figure 4C). Meanwhile, the Rh B intensity in tumor cells was relevant to the glucose concentration in Dulbecco's modified Eagle medium (DMEM) for the GC@cPt ZIF nanomotor group (Figure 4D and Figure S16, Supporting Information), further giving us evidence that glucose was fueled to propel the motion of GC@cPt ZIF nanomotor at this stage.

Next, trans‐well migration and 3D multicellular tumor spheroids (MTSs) experiments were further developed to evaluate the penetration ability of GC@cPt ZIF motor (Figure 4E,G). In trans‐well model, the lower compartment was seeded with 4T1 cell, which was isolated from the upper compartment by an insert trans‐well chamber. As shown in Figure 4F, the enzyme‐engineered GC@cPt ZIF motor group exhibited better penetration effect to tumor cells via long‐distance transport, which may be caused by the self‐propulsion of the nanomotors. We thought it might be the reason that the improved “motion ability” rather than the *Brownian* movement can facilitate the deep tumor penetration across the dense collagen fibers in tumor tissue. In 3D MTSs model, it can be clearly observed from the *z*‐stacking confocal laser scanning microscopy (CLSM) images that the GC@cPt ZIF motor group exhibited a deep penetration than nonmotor group (Figure 4H). The red fluorescence intensity in motor group is much higher than that of nonmotor group at the same condition, even at a 100 µm penetration depth. The quantitative fluorescence intensity in the 100 µm depth of the MTSs in Figure 4H also indicated this trend. For GC@cPt ZIF motor group, both the penetration depth and the average in‐depth intensity were found to be much higher than that of the nonmotor group (Figure 4I). All these results demonstrated that the self‐propelled GC@cPt ZIF motors can facilitate the penetration capability and accumulation in tumor tissues.

### 2.5 In Vitro Synergistic Tumor Therapy of GC6@cPt Motors

**Figure 5:**
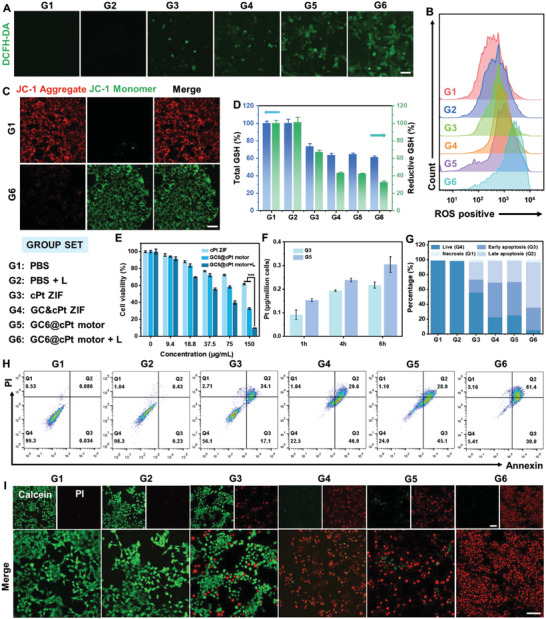
In vitro synergistic antitumor therapy of self‐propelled GC6@cPt motors. A) CLSM images and B) flow cytometry analysis of 4T1 cells stained with DCFH‐DA to reflect the intracellular ROS level after being treated with PBS (G1), PBS + L (G2), cPt ZIF (G3), GC&cPt ZIF (G4), GC6@cPt motor (G5), and GC6@cPt ZIF motor + L (G6). C) JC‐1 staining of 4T1 cells after treated with PBS (control) and GC6@cPt ZIF motor group. D) Intratumoral secretion of total GSH (left, GSH+GSSG) and reductive GSH (right) content after different formulations. E) Relative viability of 4T1 cells cultured in high glucose DMEM (4.5 g L^−1^) when treated with cPt ZIF, GC6@cPt ZIF motors, and GC6@cPt ZIF motors upon laser irradiation at different nanoparticle concentrations. The irradiation density was 100 mW cm^−2^ at 660 nm, and the irradiation time was 5 min. F) Cellular uptake of Pt content upon treatment with nonmotor (G3) and nanomotor (G5) for 1, 4, and 6 h. Data represent mean ± SD (*n* = 3). G) Quantitative analysis of live cells and apoptosis degree of the results in (H). H) Flow cytometry analysis of 4T1 cells using Annexin V‐FITC and PI staining and I) live/dead staining images of 4T1 cells using calcein‐AM (green) and PI (red) test kits treated with different formulations. Scale bar is 100 µm. Data represent mean ± SD (*n* = 5). *p < 0.05, **p < 0.01, ***p < 0.001.

Such progress promoted us to think whether it can enhance the synergistic antitumor therapy. Herein, a murine mammary carcinoma cell line 4T1 was used as a model to evaluate the anticancer activity of the GC6@cPt motors. In order to give more detailed classification, we further set up six groups: PBS (G1), PBS + L (G2), cPt ZIF (G3), nonmotor group GC&cPt ZIFs (G4), GC6@cPt ZIF motor (G5), and GC6@cPt ZIF motor + L (G6), to investigate the in vitro synergistic tumor therapy of the GC6@cPt motors. The cellular generation of ^1^O_2_ by GC6@cPt ZIF motor after cell uptake upon laser irradiation (660 nm, 100 mW cm^−2^) was quantitatively detected by using a fluorescence probe 2ʹ,7ʹ‐dichlorofluorescein diacetate (DCFH‐DA), which could convert into 2ʹ,7ʹ‐dichlorofluorescein (DCF) with strong green fluorescence in the presence of ^1^O_2_ species.^[^ ^46^ ^]^ Because the therapeutic cPt drugs can activate intracellular nicotinamide adenine dinucleotide phosphate oxidases to generate superoxide,^[^ ^31^ ^]^ negligible green fluorescence was observed inside the cells when cultured with cPt ZIF (G3) or GC6@cPt motor (G5). However, strong green signal was visible upon laser irradiation for 5 min in the presence of GC6@cPt motor (G6) (**Figure** **5**A,B). The enhancement of the green fluorescence might attribute to the PDT‐induced generation of ^1^O_2_.

As we know, the continuous accumulation of oxidative species and depletion of antioxidative GSH would disrupt the mitochondrial redox homeostasis, which seriously affected many biological processes like energy supply, and regulating mitochondrial function.^[^ ^47^ ^]^ JC‐1 probe was first exploited to investigate changes in mitochondrial membrane potential (MMP). It showed strong red fluorescence and weak green fluorescence with the ratio (*r*) of green/red nearly 0 for 4T1 cells when treated with control group (G1). However, after being cultured with GC6@cPt ZIF nanomotors for 4 h and laser irradiation (G6), more JC‐1 monomers were formed in mitochondria and an increased green fluorescence with *r* value of about 14 was visible, which indicated the process of cell apoptosis was happened (Figure 5C and Figure S17, Supporting Information). While then, the glucose concentration, reductive GSH, and total GSH content in the cells were investigated after different formulations (Figure 5D and Figure S18, Supporting Information). In comparison with the control group (G1), extracellular or intracellular glucose, total GSH, and reductive GSH were furthest reduced to the 25%, 61%, 34%, and 61%, respectively, when 4T1 cells were treated with GC6@cPt ZIF motor upon the laser irradiation (G6). Meanwhile, the generation of ROS species could direct react with reductive GSH for depletion, on the other hand, the oxidative stress in tumor cells could downregulate the production of GSH species, which further restricted the generation of reductive GSH. Interestingly, the depletion of reductive GSH could reduce the detoxification of cPt drugs by decreasing the generation of cPt‐GSH adducts, which facilitating the chemotherapeutic efficacy.^[^ ^31^ ^]^

Thiazolyl blue tetrazolium bromide (MTT) assay indicated that the laser‐triggered nanomotor group (GC6@cPt motor + L) showed much high cytotoxicity for 4T1 tumor cells in high glucose (4.5 g L^−1^) DMEM medium than cPt ZIF group and GC6@cPt ZIF motor group (Figure 5E). It was found that the glucose consumption by GC6@cPt ZIF motors could cause the insufficient supply of nutrition for starvation therapy, on the other hand, it also enhanced the type II PDT and sensitivity of the chemotherapeutic cPt through GSH depletion (shown in Scheme 1B). Interestingly, ICP‐OES was found that the nanomotor group (G5) exhibited significantly higher Pt content (0.22 µg Pt per million cells) in 4T1 cells than that (0.22 µg Pt per million cells) of nonmotor group (G3) after 4 h incubation (Figure 5F), which indicated that the nanomotor might facilitate the penetration and uptake behavior for improved cancer therapy. For comparison, such synergistic effects were not that distinguishable in low glucose (1.5 g L^−1^) DMEM (Figure S19, Supporting Information). Not only that, it was also found that the cPt ZIF scaffolds exhibited relatively lower cytotoxicity to 3T3 cells than 4T1 cells (Figure S20, Supporting Information), while the laser‐triggered GC6@cPt ZIF nanomotor (G6) also exhibited significant high cytotoxicity to both Melanoma B16 cells and human cervical Hela cells (Figure S21, Supporting Information). These results further confirmed that the laser‐irradiated GC6@cPt motor (G6) had superior synergistic anticancer efficacy. The flow cytometry results indicated that the apoptosis proportion of laser‐irradiated motor (G6) was 91.4%, while it was only 0.68%, 41.2%, and 74% for laser (G2), cPt (G3), and nonlaser group (G4), respectively (Figure 5G,H). The synergistic effect was then investigated by the live/dead co‐staining analysis using calcein AM/propidium iodide (PI), as well as by flow cytometry staining with Annexin V‐FITC and PI. Distinct cell death occurred in live/dead co‐staining for G6 group because of the synergistic effect of starvation, chemo‐, and photodynamic therapy, compared with others groups (Figure 5I).

### 2.6 In Vivo Synergistic Antitumor Efficiency

**Figure 6:**
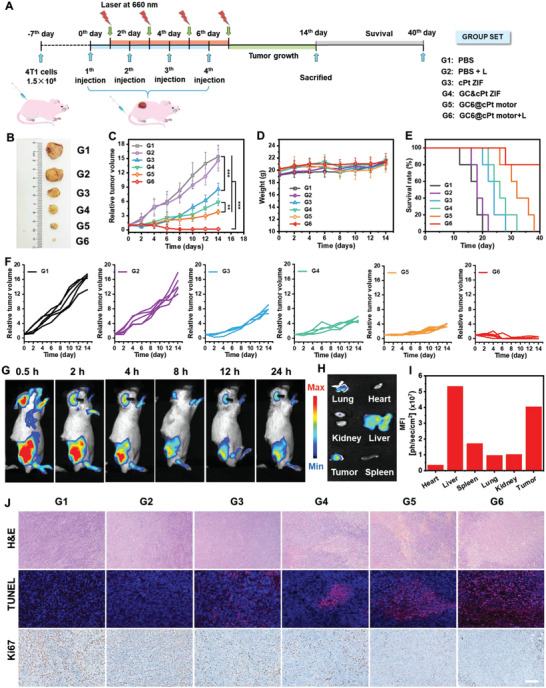
In vivo synergistic antitumor therapy of self‐propelled GC6@cPt ZIF nanomotors. A) Schematic illustration of GC6@cPt ZIF motors therapy on a 4T1‐bearing mouse model. The mice were administered intravenously with PBS (G1), PBS + L (G2), cPt ZIF (G3), GC&cPt ZIF (G4), GC6@cPt ZIF motor (G5), and GC6@cPt ZIF motor + L (G6) on 0, 2nd, 4th, and 6th day, tumor volume and survival rate were recorded on 14th and 40th day, respectively. B) Photograph of isolated 4T1 tumors of mice on the 14th day. C) Tumor growth curves, D) body weights, E) Kaplan–Meier survival plots of mice, and F) tumor growth curves of each mice after different formulations (*n* = 5). The irradiation density was 200 mW cm^−2^ at 660 nm, and the irradiation time was 10 min. G) In vivo bioluminescence images of the mice at different time points to track the distribution of GC6@cPt ZIF motor. H) Ex vivo image and I) the quantified relative fluorescence intensity of the main organs (heart, liver, spleen, lung, and kidney) and tumor isolated from 4T1 tumor‐bearing mice at 24 h post‐injection of GC6@cPt motors. J) H&E, TUNEL, and Ki67 staining of tumor tissues collected from the mice administrated with various formulations. Scale bar is 100 µm. Data are shown as mean ± S.D. (*n* = 5). **p < 0.01, ***p < 0.001.

Encouraged by the above outstanding in vitro results, in vivo synergistic anticancer effects of GC6@cPt motors were investigated on tumor‐bearing mice model. As schematically illustrated in **Figure** **6**A, the 4T1 tumor‐bearing mice model was created by direct subcutaneous implantation of nearly 1.5 × 10^6^ 4T1 tumor cells per BALB/c nude mice at −7th day. When the tumor volume reached 70 mm^3^, the 4T1 tumor‐bearing mice were randomly divided into six groups and treated with PBS (G1), PBS + L (G2), cPt ZIF (G3), GC&cPt ZIF (G4), GC6@cPt ZIF motor (G5), and GC6@cPt ZIF motor + L (G6) by intravenous injection on the 0th, 2nd, 4th, and 6th day (10 mg kg^−1^), respectively. The tumors were exposed to 660 nm laser irradiation at 16 h after i.v. injection for PDT. After treatment for 14 days, it shows the visual differences for the tumor structures (Figure 6B). The PBS‐treated tumor (G1) exhibited a 15.4‐fold increase of tumor volume, implying the fast growth and malignance of tumor cells. In comparison, the only laser treatment (G2) and cPt ZIF (G3) treatment demonstrated antitumor ability with 14.6‐fold and 8.6‐fold increase. Compared with nonmotor GC&cPt ZIF group (G4), GC6@cPt ZIF motor (G5) with similar contents exhibited only 3.78‐fold increase versus 5.83‐fold in tumor volume because of the motor‐propelled penetration efficacy and accumulation behavior. Such level can be significantly decreased to 0.2‐fold increasement when treated with GC6@cPt motors under laser irradiation (G6) (Figure 6C,F). The combination of glucose consumption, oxygen‐generation enhanced PDT, and GSH‐downregulation enhanced chemotherapy of cPt significantly facilitates the outstanding therapeutic outcome in cancer therapy.

Kinetic monitoring of the mice body weight showed no obvious body weight loss during 14 days of treatment, indicating low adverse effects of these nanoparticles to the healthy organs (Figure 6D). Consistent with the results of tumor growth inhibition, survival analysis from the Kaplan–Meier plots showed that survival time of mice has been significantly prolonged in G6 group compared with others. Specifically, the G6 group could effectively prolong the survival of mice and achieve a highest 80% survival ratio after 40 days evaluation, while all the deaths (0% survival) were found for others groups (from G1 to G5) (Figure 6E). To evaluate the in vivo pharmacokinetics, sulfo‐cyanine5.5 NHS ester (Sulfo‐Cy5.5‐NHS) was used to label enzymes to prepare sulfo‐Cy5.5‐labeled GC6@cPt ZIF nanomotors. Whole body fluorescence image, ex vivo fluorescence microscopy, and the quantified relative fluorescence intensity of main organs and tumor indicated that GC6@cPt ZIF motors were rapidly and significantly accumulated at tumor sites after 24 h post‐administration (Figure 6G–I and Figure S22, Supporting Information). These results demonstrated that integrated deep penetration of nanomotors and enhanced antitumor therapy of chemotherapy/PDT ablated the tumor completely.

Furthermore, hematoxylin and eosin (H&E) staining revealed that GC6@cPt ZIF motors upon laser irradiation (G6) showed severe tissue necrosis and nuclear pyknosis across a large tumor area after 14 days treatment, while little different was observed for control groups (Figure 6J). Besides, the serious apoptotic damage (purple spots) and the lowest proliferation (brown spots) of tumor tissues were, respectively, detected from the terminal deoxynucleotidyl transferase‐mediated dUTP nick end‐labeling (TUNEL) and Ki67‐positive immunohistochemical staining for G6 groups, demonstrating significant anticancer ability of nanomotors in vivo. Despite all this, the GC6@cPt ZIF motors even upon laser irradiation showed negligible effects to the main organs (heart, liver, spleen, lung, and kidney) of mice after treated for 14 days (Figure S23, Supporting Information). Meanwhile, the GC6@cPt ZIF nanomotors exhibited little hemolysis rate (Figure S24, Supporting Information) and reasonable blood glucose fluctuation (Figure S25, Supporting Information), indicating their excellent biocompatibility and biosafety as drug delivery platform for cancer therapy.

## 3 Conclusion

In summary, an efficient self‐propelled GC6@cPt ZIF nanomotor with self‐cascade catalysis and self‐supplied O_2_ was developed based on the cisplatin prodrug platform for boosting multimodel synergetic cancer therapy efficiency. The fabricated GC6@cPt ZIF nanomotor can be fueled by glucose in medium to produce gaseous O_2_ with enzymatic cascade reactions, which can not only propel the self‐motion of motor in tumor sites for deep penetration and high accumulation, but also enriched the raw materials of ^1^O_2_ for O_2_‐dependent PDT. Within the TME, GC6@cPt ZIF nanomotor was collapsed to release the encapsulated GOx, CAT, Ce6, and skeletal cPt drug in response to their low pH and overexpressed GSH. Subsequently, improved GOx and CAT activities through exhausting glucose enable the enhancement of photodynamic and chemo therapy by accelerating the O_2_ generation, oxidative stress, and amplifying the downstream GSH depletion, meanwhile endowed with starvation therapeutic ability, leading to intensified in situ DNA disruption and cell apoptosis. We envision that this paradigm of intelligent enzyme‐engineered cisplatin nanomotor can be extended to a variety of external fields with new functions from inside out. Moreover, this work also highlights promising prodrug‐skeleton nanomotors for intensive synergistic therapy against cancer as well as other diseases.

## 4 Experimental Section

### Synthesis of Imidazole‐Terminated Cisplatin Prodrug (cPt‐IM)

The cPt‐IM was synthesized by PyBOP‐catalyzed amidation reaction of cPt‐COOH and *N*‐(3‐aminopropyl) imidazole. In general, the cPt‐COOH (267 mg, 0.5 mmol), PyBOP (572 mg, 1.1 mmol), and diisopropylethylamine (DIEA, 182 µL, 1.1 mmol) were dissolved in anhydrous dimethylformamide (10 mL). Then, the *N*‐(3‐aminopropyl) imidazole (125 mg, 1.0 mmol) was added into the solution and the mixture was stirred at room temperature under a nitrogen atmosphere for 24 h. The crude product was collected using a rotary evaporator and further purified to get cisplatin prodrug imidazole (cPt‐IM). Yield: 64%.

### Fabrication of GC6@cPt ZIF Nanomotors

The detailed fabrication procedure of GC6@cPt ZIF nanomotors was similar to the procedure of cPt ZIFs. Specially, 1 mL methanol/water solution (1:6) of glucose oxidase (GOx, 133 µg mL^−1^), catalase (CAT, 66.7 µg mL^−1^), and Chlorin e6 (Ce6, 50 µg mL^−1^) was mixed with cPt‐IM (20 mg, 26.8 mmol) under stirring. 1 mL Zn(NO_3_)_2_·6H_2_O solution (1.3 mg, 4.5 mmol) was added into the mixture in the presence of amphiphilic block copolymer DSPE‐mPEG2000. The mixture solution was further stirred for 2 h and then washed at least three times with ultrapure water. The GC6@cPt ZIF nanomotors were then lyophilized and appeared pink in brown (2.8 mg), which were stored at 4 °C. Similarly, G@cPt ZIFs, C@cPt ZIFs, and GC@cPt ZIFs were also constructed using the same ZIF formation procedure with only GOx, CAT, and GOx/CAT existed, respectively.

### Evaluation of Encapsulation Efficiency and the Loading Percentage

The cPt amount, enzyme concentration, and Ce6 in the GC6@cPt ZIF nanomotors were measured by ICP‐OES, BCA assay, and UV‐vis measurements, respectively, using their own standard curves. The encapsulation efficiency (EE) and the loading percentage (LP) of each component in GC6@cPt ZIF nanomotors were determined as follows

(4) $$\mathrm{EE}\;\left(i,\%\right)=\frac{m_{i}\left(\mathrm{in}\;\mathrm{GC}6@\mathrm{cPt}\right)}{m_{i,o}}\;\times 100\%$$

(5) $$\mathrm{LP}\;\left(i,\%\right)=\frac{m_{i}\left(\mathrm{in}\;\mathrm{GC}6@\mathrm{cPt}\right)}{m\left(\mathrm{GC}6@\mathrm{cPt}\right)}\;\times 100\%$$

where *m_i_*, *m*_*i*,*o*_, and *m* are the mass values of testing component in GC6@cPt ZIFs, initially added component when making GC6@cPt ZIFs, and total GC6@cPt ZIFs, respectively. As detected, the EE and LP of total GOx and CAT enzymes were measured to be 71.7% and 5.1%, respectively. The LP values of cPt drugs and Ce6 were measured to be 12.63% and 1.3%, respectively.

### Evaluation of Enzyme Stability

The enzyme stability of nanomotors was evaluated with the generation of H_2_O_2_ utilizing TMB/HRP as detection agent. Samples were mixed in glucose solution in the presence of TMB/HRP color metric probes for 10 min to quantitatively evaluate the enzymatic activity with a characteristic absorption peak at 652 nm for the oxidative TMB. The activity of fresh sample was defined as 100%. For the relative enzyme activity of each time, the same operations were carried out with the remaining reaction solution to refer the stability of enzymes in nanomotors.

### Evaluation of Motion Behavior

The movement of nanoparticles was recorded by inverted microscope with a 63 × oil objective. The G@cPt ZIFs, C@cPt ZIFs, and GC@cPt ZIFs were, respectively, dispersed in glucose solution with different concentration and placed in a glass slide with a cover. The movement of nanoparticles was imaged in a bright field, and the trajectory of particles was analyzed by Image J software. The mean‐squared displacement (MSD) was calculated as following

(6) $$\begin{array}{ccc} \mathrm{MSD}\left(\Delta t\right) & = & \langle {\left(x_{i}\left(t+\Delta t\right)-x_{i}\left(t+\Delta t\right)\right)}^{2}\rangle \;\;\\ & & \;\left(i=2,\;\mathrm{for}\;2D\;\mathrm{analysis}\right) \end{array}$$

### Cellular Uptake of GC6@cPt ZIF Nanomotors

Cellular uptake was measured by CLSM. Briefly, 4T1 cells were first seeded in 8 well plates and incubated at 37 °C for 24 h, then the cells were incubated with DMEM medium containing RhB‐labeled GC6@cPt ZIFs of different glucose concentration (1, 3, 4.5 g L^−1^) for 4 h. Then the DMEM was removed and cells were washed with PBS for three times, and added with Hoechst 33342 for staining. After 20 min, the cells were washed with PBS again for further observation by CLSM. Nonmotor group was treated with DMEM medium containing RhB‐labeled G@cPt ZIFs as control. The fluorescence intensity value is processed by Image J software.

### Penetration Ability of GC6@cPt ZIF Nanomotors

The deep permeation ability of nanomotors was evaluated by both transwell and 3D multicellular tumor spheroids (3D MTS) model. In short, a cell climbing plate was placed in the basal chamber of a transwell with polycarbonate membranes (12‐well, 0.4 µm; Corning), and 4T1 cells were seeded and incubated overnight. Then, RhB‐labeled GC6@cPt ZIFs and RhB‐labeled G@cPt ZIFs were added into the apical chamber of the transwell plate. After 4 h incubation, the cells were washed with PBS and stained with Hoechst 33342, then the cell climbing plate was taken out for detection by CLSM. To establish 3D MTS model, 4T1 cells were seeded into a 96‐well plate containing 1.5 wt% agarose, and incubated for 72 h to obtain relatively compact tumor spheres. Then, the 3D MTSs with similar size were selected and incubated with RhB‐labeled GC6@cPt ZIFs and RhB‐labeled G@cPt ZIFs for 8 h to evaluate the tumor penetration ability of the motors, and also observed by CLSM.

### Determination of Cellular ROS

ROS generation in cells was measured by using the standard probe DCFH‐DA. Briefly, 4T1 cells were seeded in 8‐well plate at a density of 5 × 10^5^ per well with DMEM containing 10% fetal bovine serum (FBS), 1% PS for 24 h at 37 °C. Then, the cells were treated with PBS, PBS+L, cPt ZIFs, GC&cPt ZIFs, GC6@cPt ZIFs, and GC6@cPt ZIFs+L, respectively, and incubated for another 4 h. For the laser irradiation, the samples were exposed to 660 nm laser with 100 mW cm^−2^ for 5 min. After further incubation with blank DMEM containing DCFH‐DA for 30 min, the DMEM was removed and cells were washed with PBS for three times. The fluorescence imaging of cells was analyzed by CLSM.

### In Vitro Mitochondrial Function Assay

MMP was detected by the enhanced mitochondrial membrane potential assay kit with JC‐1 fluorescent probe. In short, 4T1 cells were seeded in 8‐well plates and incubated at 37 °C for 24 h, then the cells were treated with GC6@cPt ZIFs, or without nanoparticles (control) and further incubated for 4 h. Then both groups were exposed to 660 nm laser with 100 mW cm^−2^ for 5 min. Afterward, the DMEM was removed and cells were cultured at 37 °C for 20 min with JC‐1 staining solution. After washing with JC‐1 buffer for two times, the cells were detected by CLSM.

### Cytotoxicity of GC6@cPt ZIF Nanomotors

4T1 murine breast cancer cells were cultured in DMEM medium containing 10% FBS and 1% penicillin/streptomycin in a 5% CO_2_ incubator at 37 °C. The cytotoxicity was evaluated against 4T1 cells using MTT assay. Briefly, 4T1 cells were seeded in 96‐well plates at a cell density of 1 × 10^4^ cells per well, and incubated for 24 h, followed by rinsing with PBS and incubation for another 24 h in fresh DMEM medium containing GC6@cPt motors at varied cPt concentrations (0, 0.37, 0.73, 1.45, 2.9, and 5.8 µg mL^−1^). After incubation for 4 h, the cells were washed by PBS, followed by laser irradiation (660 nm, 100 mW cm^−2^) for 5 min. For comparison, cPt ZIFs and GC6@cPt nanomotors without laser irradiation were used as control. After incubation for 20 h, the cells were added with 10 µL of MTT reagent and incubated for 4 h at 37 °C. The absorbance at 560 nm was measured to calculate the cell viability that was normalized by the control group (100%) without any treatment.

### Live/Dead Cell Staining

The live/dead cell staining was carried out using calcein/PI live/dead viability/cytotoxicity assay kit according to published method. In brief, 4T1 cells (5 × 10^4^ cells per confocal dish) were cultured at 37 °C for 24 h. After removing the culture medium, the cells were incubated with fresh DMEM medium containing PBS, cPt ZIFs, GC&cPt ZIFs, and GC6@cPt ZIF nanomotors for 4 h, followed by treating with or without 660 nm laser irradiation (100 mW cm^−2^) for 5 min, and further cultured for another 4 h. After that, calcein AM (2 × 10^−6^ m) and PI (2 × 10^−6^ m) were added, and then washed with fresh medium twice for CLSM. The excitation wavelength was 488 nm for calcein AM and 532 nm for PI, respectively.

### Cell Apoptosis Assays

Annexin V‐FITC/PI was used to analyze the cell apoptosis by flow cytometry. 1 × 10^6^ 4T1 cells per well were seeded in a 6‐well plate for 24 h. Then, incubated with PBS, cPt ZIFs, GC&cPt ZIFs, and GC6@cPt ZIFs for 4 h, followed by treating with or without 660 nm laser irradiation (100 mW cm^−2^) for 5 min, and further cultured for another 4 h. Then, both live and dead cells were collected and added with Annexin V‐FITC/PI. After incubation for 20 min in dark, the cell apoptosis was analyzed by flow cytometry.

### In Vivo Fluorescence Imaging

All animal experiments were carried out in the Laboratory Animal Center of Hangzhou Normal University and were approved by the Ethics Committee of Laboratory Animal Center, Hangzhou Normal University. In general, for the 4T1 murine breast tumor model, 4T1 tumors were hypodermic inoculation through subcutaneous injection of 4T1 cells (2 × 10^6^ cells) in the lateral region of the back of BALB/c mice. When the tumor volume reached 200 mm^3^, 100 µL of free Cy5.5 or Cy5.5‐labeled GC6@cPt ZIFs (0.1 mg mL^−1^) was i.v. injected into 4T1 tumor‐bearing mice (*n* = 3). The in vivo fluorescence images of mice at 0.5, 2, 4, 8, 12, and 24 h were carried out using an IVIS Spectrum imaging apparatus with a 676/694 nm excitation/emission filter. At 24 h, the mice were sacrificed under anesthesia and the tumors and major organs (heart, liver, spleen, lung, and kidney) were collected for ex vivo imaging. Meanwhile, the fluorescence intensity in each tissue was also recorded.

### In Vivo Antitumor Efficiency

The 4T1 tumor‐bearing BALB/c mice were established using the aforementioned method. The tumor‐bearing mice were used for subsequent antitumor treatment until the tumor volume reached about 70 mm^3^. Relative tumor volumes were calculated as *V*_t_/*V*_0_ (*V*_0_ is the tumor volume at 0th day). 4T1 tumor‐bearing mice were randomly divided into six group (5 mice per group) and, respectively, treated with different formula via tail intravenous injection: PBS (G1), PBS + L (G2), cPt ZIF (G3), GC&cPt ZIF (G4), GC6@cPt motor (G5), and GC6@cPt motor + L (G6). After injection for 16 h, laser groups were exposed to laser (200 mW cm^−2^, 660 nm) for 10 min. Each group was performed every other day for a total of four times at the first few days. The tumor size and body weight were measured and recorded every other day by using digital calipers and an electronic balance. After the 14th day treatment, the mice were sacrificed and their tumor tissues were collected for photographing. To further histological analysis, harvesting major organs (hearts, lungs, livers, spleens, and kidneys) and tumors were observed for H&E, TUNEL, and Ki67 staining. Mice survival was independently assessed over a period of 40 days.

## Conflict of Interest

The authors declare no conflict of interest.

## Supporting information

Supporting Information

## Data Availability

The data that support the findings of this study are available in the Supporting Information of this article.
