# Supplementary material for: Self‐Propelled Enzymatic Nanomotors from Prodrug‐Skeletal Zeolitic Imidazolate Frameworks for Boosting Multimodel Cancer Therapy Efficiency
Source: Adv Sci (Weinh). 2023 May 15;10(22):2301919. doi: 10.1002/advs.202301919 (PMC10401186; doi:10.1002/advs.202301919)
Supplement: Supplementary file 1 — Supporting Information [file ADVS-10-2301919-s001.pdf]

ADVANCED SCIENCE
Open Access

Supporting Information

for *Adv. Sci.*, DOI 10.1002/advs.202301919

Self-Propelled Enzymatic Nanomotors from Prodrug-Skeletal Zeolitic Imidazolate Frameworks for Boosting Multimodel Cancer Therapy Efficiency

Jieyu Yu, Yan Li, An Yan, Yuwei Gao, Fei Xiao, Zhengwei Xu, Jiayun Xu, Shuangjiang Yu, Junqiu Liu and Hongcheng Sun**

((Supporting Information can be included here using this template))

Supporting Information

Self-propelled Enzymatic Nanomotors from Prodrug-skeletal Zeolitic Imidazolate Frameworks for Boosting Multimodel Cancer Therapy Efficiency

Jieyu Yu,[#] Yan Li,[#] An Yan,[#] Yuwei Gao, Fei Xiao, Zhengwei Xu, Jiayun Xu, Shuangjiang Yu, Junqiu Liu and Hongcheng Sun**

College of Material, Chemistry and Chemical Engineering, Key Laboratory of Organosilicon Chemistry and Material Technology, Ministry of Education, Key Laboratory of Organosilicon Material Technology of Zhejiang Province, Hangzhou Normal University, Hangzhou 311121, Zhejiang, P. R. China.

E-mail: junqiuliu@jlu.edu.cn; sunhc@hznu.edu.cn

Experimental Section

1. Chemicals and Materials

Succinic anhydride, hydrogen peroxide solution (H_2O_2, wt.30%), diisopropylethylamine (DIEA) benzotriazol-1-yl-oxytripyrrolidinophosphonium (PyBOP), N-(3-aminopropyl) imidazole, glucose oxidase (GOx), catalase (CAT), horseradish peroxidase (HRP), glucose, and 3,3',5,5'-tetramethylbenzidine (TMB) were purchased form Aladdin Reagent. Glutathione (GSH), zinc nitrate, hexahydrate ($Zn(NO_3)_2·6H_2O$), chlorin e6 (Ce6), 1,3-diphenylisobenzofuran (DPBF), 2,2,6,6-tetramethylpiperidine (TEMP), rhodamine b isothiocyanate (RhB-ITC), cyanine5.5 (Cy5.5) 1,2-distearoyl-sn-glycero-3-phosphoethanolamine-N-[methoxy(polyethylene glycol)-2000] (DSPE-mPEG2000), 3-(4,5-dimethylthiazol-2-yl)-2,5-diphenyl tetrazolium bromide (MTT) were bought from Energy Chemical. Cisplatin (cPt) was obtained from Kunming Guiyan Pharmaceutical Co., Ltd. Annexin V-FITC/PI apoptosis detection kit, calcein AM/PI double stain kit, and reactive oxygen species (ROS) assay kit (DCFH-DA) were obtained from Meilunbio. Enhanced mitochondrial membrane potential assay kit (JC-1), hematoxylin-eosin staining kit, glucose assay kit with O-toluidine, and reductive GSH and total GSH assay kit were provided by Beyotime Biotechnology. N, N-Dimethylformamide (DMF) chloroform, and methanol were purchased from Sinopharm. Group Co., LTD. (Beijing).

2. Characterizations

The ^{1}H NMR and ^{195}Pt NMR of synthesized organic molecules and their relevant derivatives were identified using Bruker DMX500 spectrometer. The molecular weight was analyzed using liquid chromatograph-mass spectrometer (LC-MS, Agilent1290-microTOF-QII). The hydrodynamic diameter (D_h), size distribution and the zeta-potential of the nanoparticles were measured by dynamic light scattering (DLS) measurements using Zetasizer Nano ZSE. The structures were characterized by OXFORD Cypher S atomic force microscopy (AFM), HITACHI HT7700 transmission electron microscope (TEM) with 100 kV accelerating voltage. Magnified structures and EDX elemental mappings were measured using FEI Tecnai G2 F20 high resolution field emission transmission electron microscope (HR-TEM) with 200 kV accelerating voltage. UV-vis spectra were recorded on a Shimadzu UV-2600 UV-vis spectrometer. Inductively coupled plasma optical emission spectrometry (ICP-OES, ICP-5000) and X-ray photoelectron spectroscopy (XPS, K-Alpha+) were used for determing the content and valency of Pt element, respectively. Electron paramagnetic resonance (EPR) was measured using Electron Spin Resonance Spectrometer (JES-X320, JEOL Ltd.). The dissolved O_2 concentration and pH value were monitored by using a portable dissolved oxygen meter

(INESA, Shanghai) and an OHAUS ST3100-F pH meter, respectively. Histochemical staining and the motion behavior of motors were measured by using Olympus IX73 inverted fluorescence microscope imaging system. Fluorescence images were observed using a confocal laser scanning microscope (CLSM, LSM 900 equipped with Airyscan 2), and flow cytometry analysis was analyzed by a BD FACS Celesta^{TM} flow cytometer. *In vivo* body fluorescent imaging and ex-vivo fluorescent imaging was obtained with a photon imagertm optima. All animal experiments were carried out in the Laboratory Animal Center of Hangzhou Normal University and were approved by the Ethics Committee of Laboratory Animal Center, Hangzhou Normal University.

3. Synthesis of cisplatin prodrug imidazole (cPt-IM)

The synthesis rout of the **cPt-IM** was shown in scheme S1.

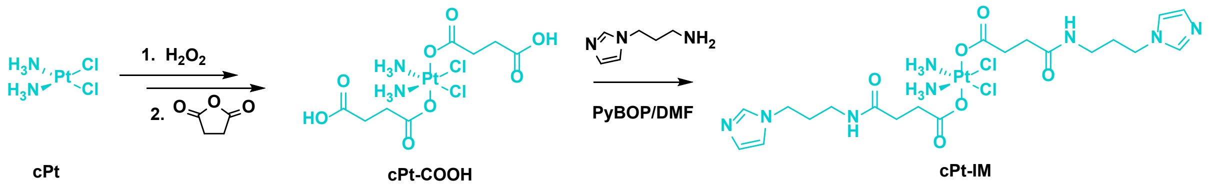

Scheme S1 Synthesis route of cPt-IM ligand

Synthesis of dicarboxyl cPt prodrug (cPt-COOH): The cPt-COOH was synthesized using modified literature procedure.[1,2] Briefly, to a 100 mL round flask, cisplatin (cPt, 600 mg, 2.0 mmol) was suspended in Milli-Q water (10 mL). Excess amount of hydrogen peroxide (20 mL, 30 wt.%) was dropped and the mixture was stirred at room temperature in the dark for 6h to get yellow solids. Then, the precipitate was collected by filtration and washed with ethanol and diethyl ether, and then dried under vacuum to yield 454 mg oxidized cPt prodrug *c,c,t*-$Pt(NH_3)_2Cl_2(OH)_2$ (cPt-OH). Yield: 68%.

After that, cPt-OH (334 mg, 1.0 mmol) and succinic anhydride (210 mg, 2.1 mmol) were dissolved in anhydrous DMF (5 mL) and the solution was stirred at 50 °C under a nitrogen atmosphere overnight. After reaction, the mixture was concentrated using a rotary evaporator and then precipitated in diethyl ether. The precipitate was collected by filtration and washed with chloroform for three times to get 294 mg dicarboxyl cisplatin prodrug *c,c,t*-$Pt(NH_3)_2Cl_2(O_2CCH_2CH_2COOH)_2$ (cPt-COOH) as yellow solid. Yield: 55%. ^{1}H NMR (500 MHz, $(CD_3)_2SO$, Figure S1): δ (ppm) = 12.07 (s, 2H, -COOH), 6.48 (m, 6H, $-NH_3$), 2.48 (t, 2H, $-CH_2$), 2.37 (t, 2H, $-CH_2$).

Synthesis of imidazole-terminated cisplatin prodrug (cPt-IM): The cPt-IM was synthesized by PyBOP-catalyzed amidation reaction of cPt-COOH and N-(3-aminopropyl)imidazole. In general, the cPt-COOH (267 mg, 0.5 mmol), PyBOP (572 mg, 1.1 mmol), and diisopropylethylamine (DIEA, 182 μL, 1.1 mmol) were dissolved in anhydrous DMF (10 mL). Then, the N-(3-aminopropyl) imidazole (125 mg, 1.0 mmol) was added into the solution and the mixture were stirred at room temperature under a nitrogen atmosphere for 24 h. The crude product was collected using a rotary evaporator and further purified to get cisplatin prodrug imidazole (cPt-IM). Yield: 64%. ^1H NMR (500 MHz, D_2O, Figure S2): δ (ppm) =8.16 (s, 1H, IM-H), 7.34 (s, 1H, IM-H), 7.23 (s, 1H, IM-H), 4.18 (d, 2H, $-NCH_2$), 3.20 (t, 2H, $-NHCH_2$), 2.68 (t, 2H, $-CCH_2C$), 2.47 (t, 2H, $-COCH_2$), 2.01 (s, 2H, $-CH_2COO$). MS (Q-TOF, m/z, Figure S3): Calc. for $C_{20}H_{34}Cl_2N_8O_6Pt$, 748.5; found: 749.2 ($[M+H]^+$; 771.1, $[M+Na]^+$; 792.6, $[M+K]^+$.

4. Fabrication of Nanomotors

Fabrication of cPt ZIFs: The detailed fabrication procedure of Pt(IV) prodrug nanoparticles (cPt ZIFs) were similar to the previously reported method.[3, 4] Briefly, cPt-IM (10 mg) was added into a vial with methanol (1 mL) under stirring. 6 μL $Zn(NO_3)_2·6H_2O$ solution (100 mg/mL) was added into the mixture and stirred for 30 min. Finally, the cPt ZIF solution was washed and concentrated by Amicon Ultra centrifugal filters (30 kDa) to remove the residual ligands.

Fabrication of GC6@cPt ZIF nanomotors: For enzyme encapsulation, a methanol/water solution (1:8) of appropriate amount of GOx, CAT and Ce6 were mixed with cPt-IM (10 mg) under stirring. 6 μL $Zn(NO_3)_2·6H_2O$ solution (100 mg/mL) was added into the mixture in the presence of amphiphilic block copolymer DSPE-mPEG2000. The mixture solution was further stirred for 30 min and then washed at least three times with ultrapure water.

Fabrication of fluorophore-labeled nanomotors: In general, rhodamine B isothiocyanate (RITC) or cyanine5.5 NHS ester (Cy5.5-NHS) were firstly co-incubated with enzymes for 4 h at 25 °C for the formation of covalent bond with amino groups on enzymes. After that, the solution was washed and concentrated by Amicon Ultra centrifugal filters (3 kDa) to remove the residual fluorophores. The fluorophore-labeled enzymes were mixed with cPt-IM and $Zn(NO_3)_2·6H_2O$ under stirring for preparation of enzyme-propelled nanomotors, with the similar procedure of GC6@cPt ZIF nanomotors.

5. Measurements

Encapsulation efficiency of enzymes and Ce6: The loading efficiency of total enzymes (GOx and CAT) was determined using BCA Protein Assay Kit. In short, the purified nanoparticles were dispersed in ultrapure water and incubated in PBS buffer (pH 5.0) for certain time, then the supernatants were collected and recorded the absorption value. The standard curve of Ce6 were plotted from the peak absorbance of standard Ce6 solution with different concentrations (1, 2, 16, 20, 40, 60, 80 μg/mL). Data was recorded with UV-vis spectra.

Performance evaluation of enzyme cascades: TMB/HRP assay was utilized as the color metric probe to quantitatively evaluate the enzymatic activity with a characteristic absorption peak at 652 nm for the oxidative TMB. Experiments were conducted in 5 mM glucose or 10mM H_2O_2 and TMB/HRP with C@cPt ZIFs, G@cPt ZIFs, and GC@cPt ZIFs, respectively. The samples collected at different time points were added with TMB and HRP successively. For accurately quantifying the H_2O_2 concentration, TMB and HRP were added into the H_2O_2 solution varied at 0.05 mM, 0.1 mM, 0.2 mM, 0.3 mM, and 0.4 mM to make the standard curve, then pending text sample was added into TMB/HRP mixtures to determine the H_2O_2 concentration.

The generation profiles of the dissolved O_2 and pH change of the solution are used to evaluate the catalytic activity of enzymes. An aqueous solution containing 100 μg/mL pending text samples was added with 5 mM glucose or 10 mM H_2O_2. The dissolved O_2 concentration and pH value of the reaction solution were monitored in real-time by a portable dissolved oxygen meter (INESA, Shanghai) and an OHAUS ST3100-F pH meter, respectively.

Evaluation of drug release: 2 mL of 2 mg mL^{-1} GC6@cPt ZIFs was added into a dialysis bag (MWCO 100 kDa) and incubated in 20 mL of PBS buffer in the presence or absence of GSH or acid solution for different intervals. At the given time points, samples were collected to detect the Ce6 content and Pt element. The release of Pt was measured by ICP-OES using a standard curve. Meanwhile, the release of Ce6 from GC6@cPt ZIFs was determined by UV–vis spectra using its own standard curve.

Detection of singlet oxygen $^1O_2$: The diphenylisobenzofuran (DPBF) was used to trap the production of $^1O_2$ of nanoparticles in different medium. For detection of GC6@cPt ZIFs + H_2O_2 + L assay, GC6@cPt ZIFs, 10 mM H_2O_2 and 25 mg mL^{-1} DPBF were incubated in 0.01 M HAc/NaAc buffer (pH = 6.2) at 37 °C. At different time points, the absorption values of samples at 420 nm were detected by a UV-vis spectrophotometer. Meanwhile, the production of $^1O_2$ was also detected by electron paramagnetic resonance spectrometer, using TEMP as a free radical trapping agent.

Detection of intracellular Pt content: 4T1 cells were seeded into 6-well plates and cultured for 24 h to reach at 1 million cells. Then, the culture medium was replaced with fresh medium containing cPt ZIFs, or GC6@cPt ZIF nanomotors with certain Pt concentration (10 μM) for 1 h, 4 h, or 6 h. The cells were collected and washed for 3 times, and intracellular Pt content was estimated by ICP-OES measurement.

Detection of cellular glucose, reductive GSH and total GSH: The content of glucose is determined by Glucose Assay Kit with O-toluidine. Briefly, the culture medium of each group was removed and the cells were washed with PBS twice, then added with lysis buffer for cytolysis. After centrifugation, supernatant (10 μL) was mixed with 180 μL Glucose Assay Agent in the PCR tube. Then, each tube was incubated in PCR instrument at 95 °C for 8 min, after then cooling down to 4 °C, 180 μL of the liquid was transferred into a clean 96-well plate and recorded the absorbance at 630 nm. The glucose concentration in the culture medium was determined in the same method. GSH and GSSG Assay Kit was used to examine the content of reductive GSH and total GSH (GSH+GSSG). For determination of total GSH, the cells was washed with PBS once, then the supernatant was removed after centrifugation and added with Protein Removal Reagent M solution. Each group was further carried out with two quick freeze-thaw treatments. After centrifugation, 10 μL supernatant was mixed with 150 μL total glutathione detection working solution and incubated at 25 °C for 5 min. Then, NADPH was added to solution and incubated for 25 min. The absorbance at 412 nm was measured and the control group was set as 100%. In order to obtain the content of reductive GSH, it is necessary to further determine the amount of GSSG. The method for determining GSSG is similar to the above, except that GSH scavenger is added to the sample of supernatant after freeze-thaw centrifugation. The calculation formula is as follows:

$$\boldsymbol{c}_{\mathrm{rGSH}} = \boldsymbol{c}_{\mathrm{tGSH}} - 2 \times \boldsymbol{c}_{\mathrm{GSSG}}$$

where $\boldsymbol{c}_{\mathrm{rGSH}}$, $\boldsymbol{c}_{\mathrm{tGSH}}$ and $\boldsymbol{c}_{\mathrm{GSSG}}$ are concentration of reductive GSH, total GSH and oxidized GSSG respectively.

Common hematology tests: 1 mL blood sample was obtained from BALB/c mice and stored with EDTA anticoagulant. The RBS was washed several times with PBS and centrifuged (3000 rpm, 10 min) at 4 ° C until the supernatant was almost clear. Then, after being mixed with 5 mL PBS, 200 μL of diluted RBC solution was then added into 800 μL of deionized water or 200 μg/mL PBS, PBS+L, cPt ZIFs, GC&cPt ZIFs, GC6@cPt ZIFs, and GC6@cPt ZIFs+L, respectively, where PBS group as a negative control and deionized water as a positive control.

After the incubation at 37 °C for 4 h, the mixture was centrifuged at 3000 rpm for 10 min, the absorbance of supernatants at 545 nm was recorded. The hemolysis percentage ($\boldsymbol{P}_{\mathrm{He}}$) of RBCs was calculated using the following equation:

$$\boldsymbol{P}_{\mathrm{He}} = \frac{\boldsymbol{A}_s - \boldsymbol{A}_n}{\boldsymbol{A}_P - \boldsymbol{A}_n} \times 100\%$$

where $\boldsymbol{P}_{\mathrm{He}}$, $\boldsymbol{A}_{\mathrm{s}}$, $\boldsymbol{A}_{\mathrm{n}}$, and $\boldsymbol{A}_{\mathrm{p}}$ refer to the hemolysis percentage, absorbance of substance, absorbance of quarantine sample, absorbance of negative control, and absorbance of positive control.

Results and Characterization

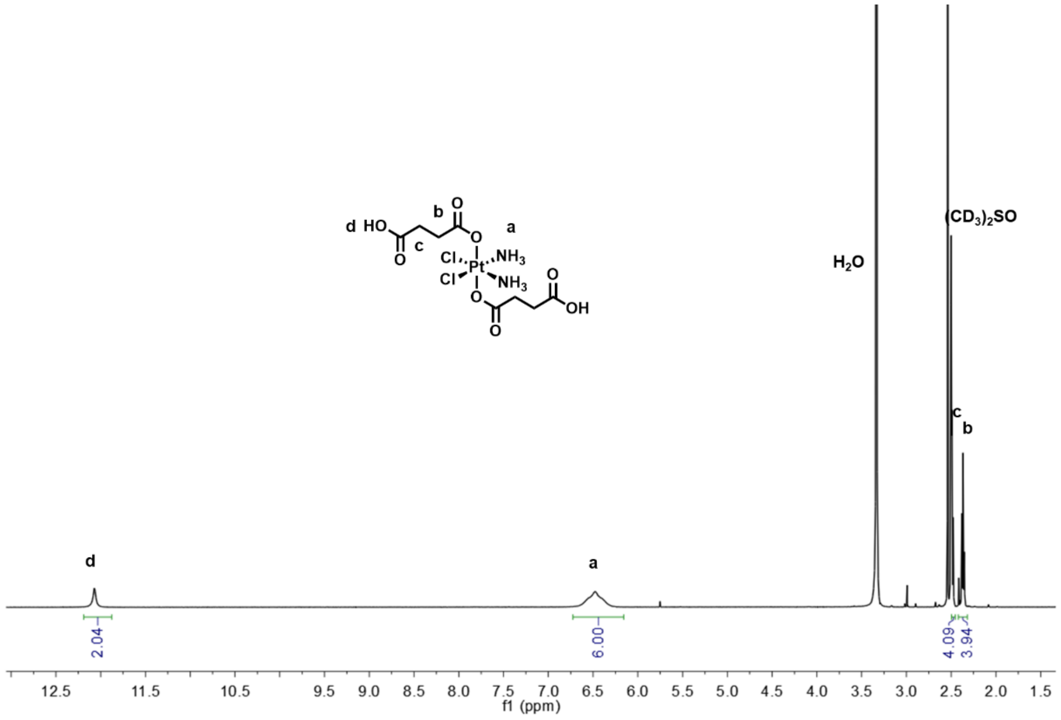

Figure S1 ^{1}H NMR spectrum of synthesized cPt-COOH in $(CD_3)_2SO$

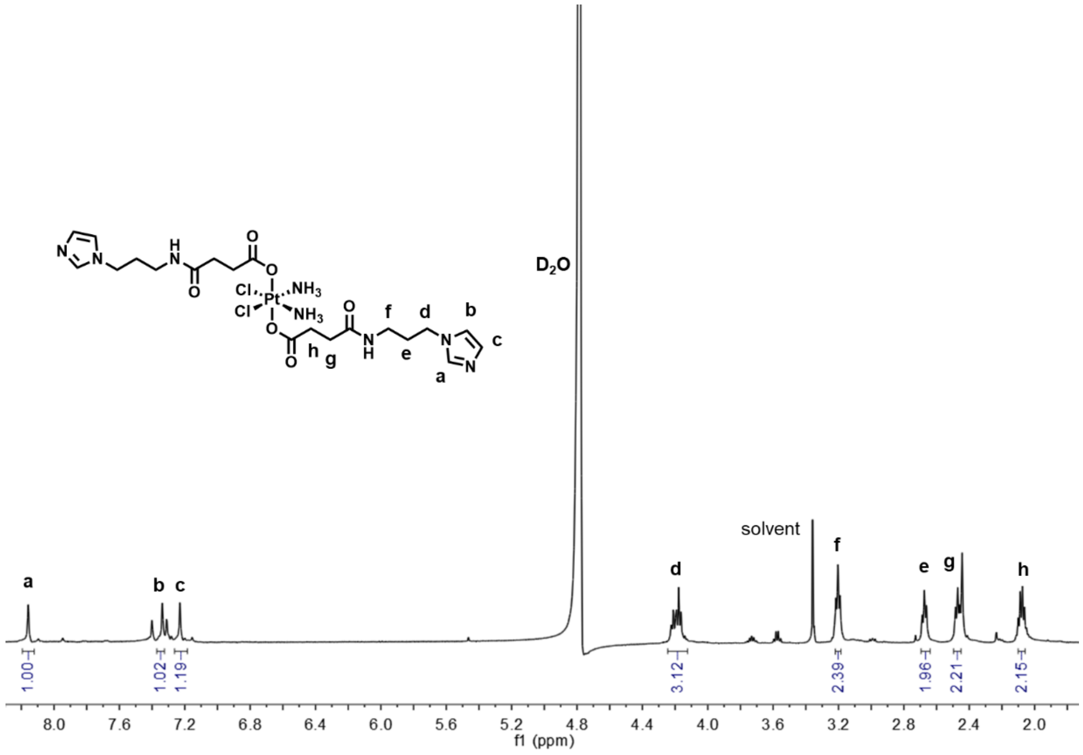

Figure S2 ^{1}H NMR spectrum of synthesized cPt-IM in D_2O

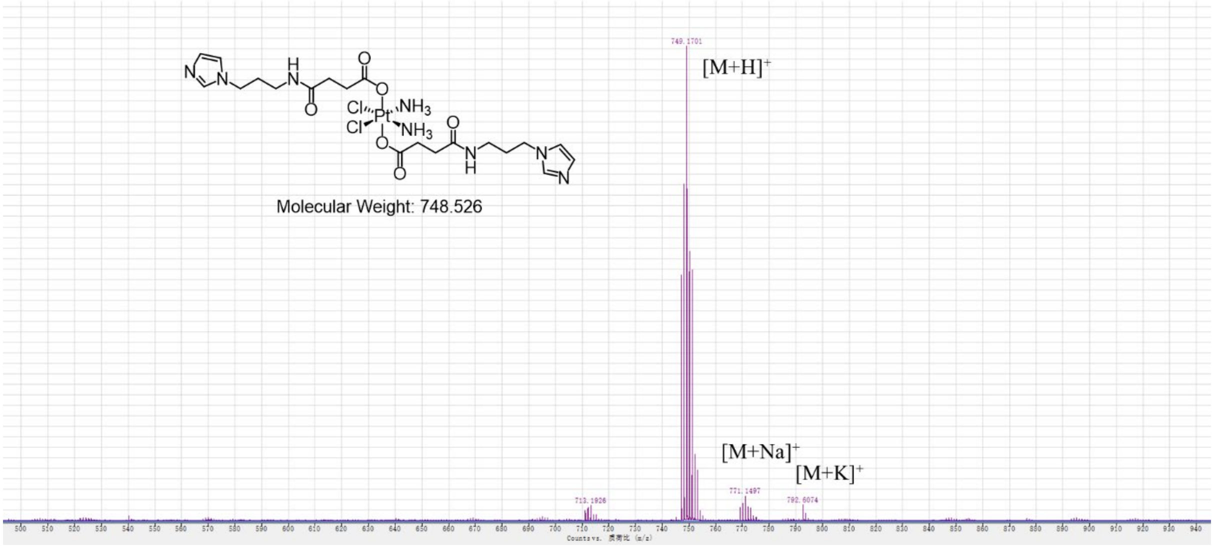

Figure S3 MS spectrum of synthesized cPt-IM.

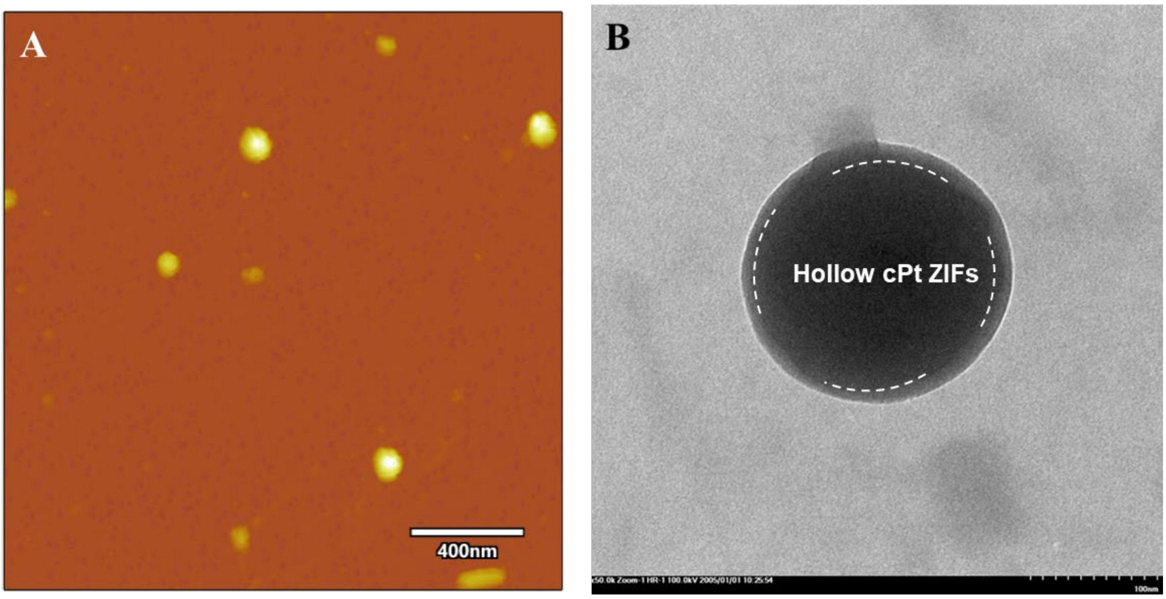

Figure S4. (A) AFM image of cPt ZIF structures. (B) TEM image of cPt ZIF structures.

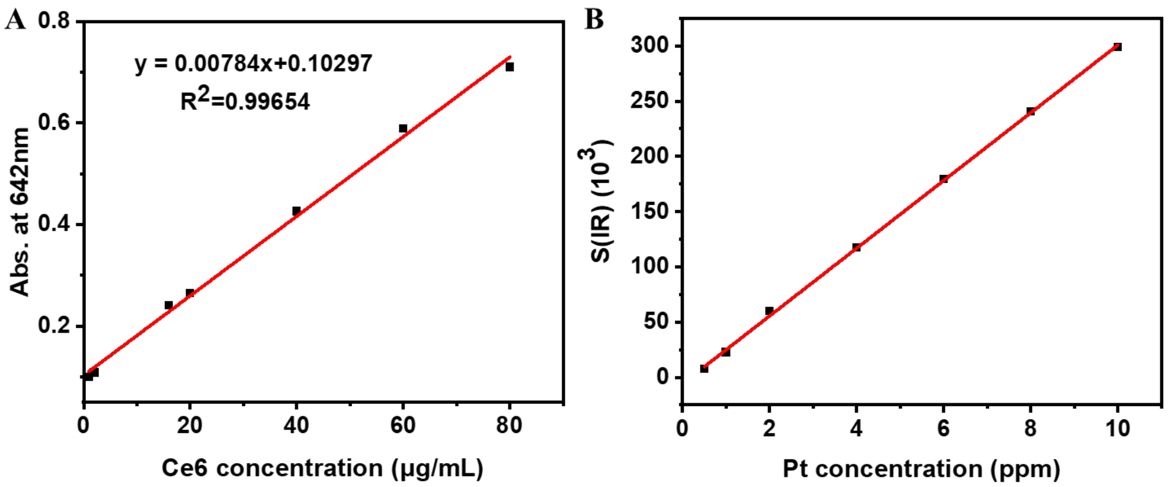

Figure S5. The standard curve of Ce6 absorption at 642 nm from UV-vis spectra. (B) The standard curve of Pt elements from ICP-OES.

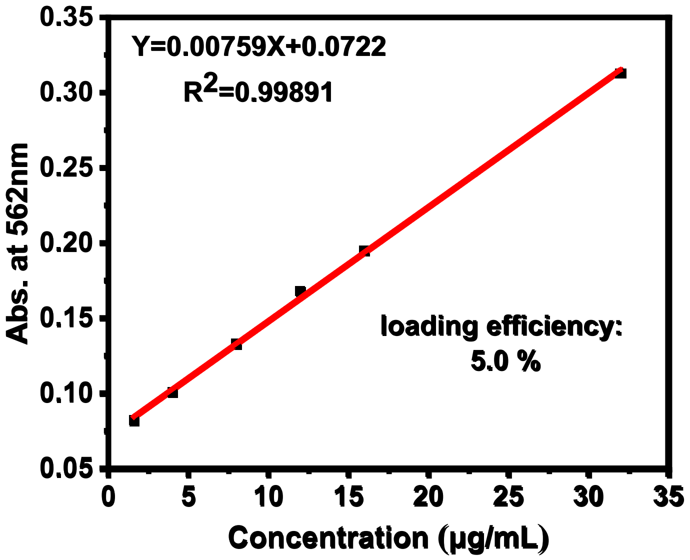

Figure S6. The standard curve of protein for BCA protein assay kit.

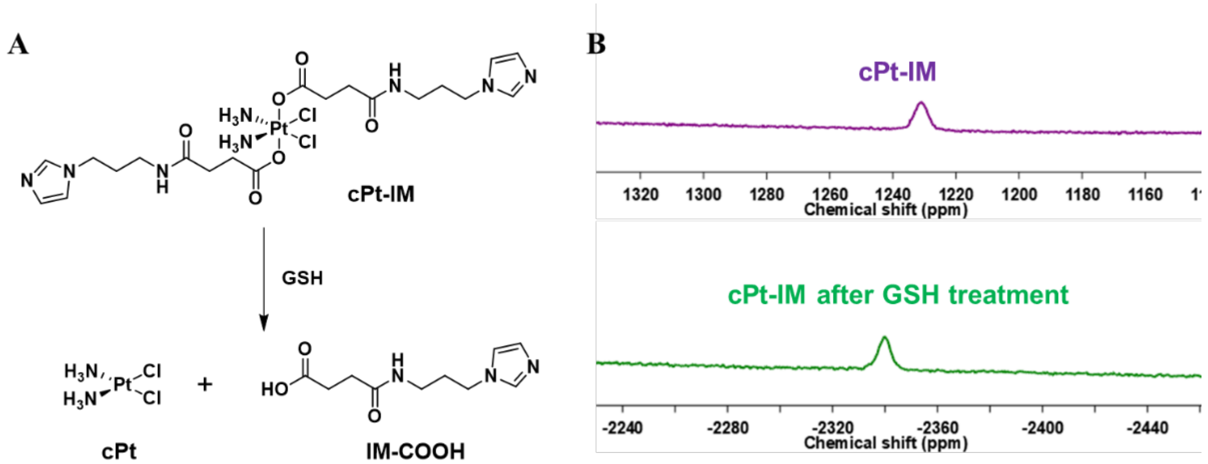

Figure S7: (A) Scheme for the reduction of cPt-IM prodrug. (B) ^{195}Pt NMR spectra of cPt-IM prodrug before and after treatment with 5 mM GSH.

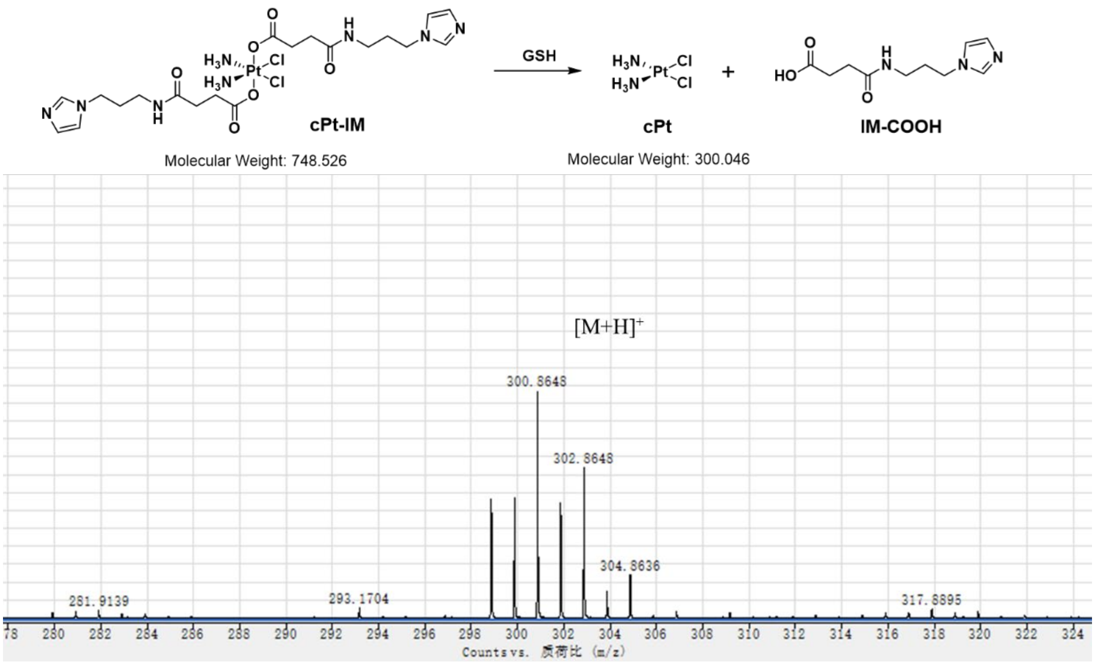

Figure S8. Reduction procedure and Q-TOF MS of cPt-IM prodrug after reduction with 5 mM GSH.

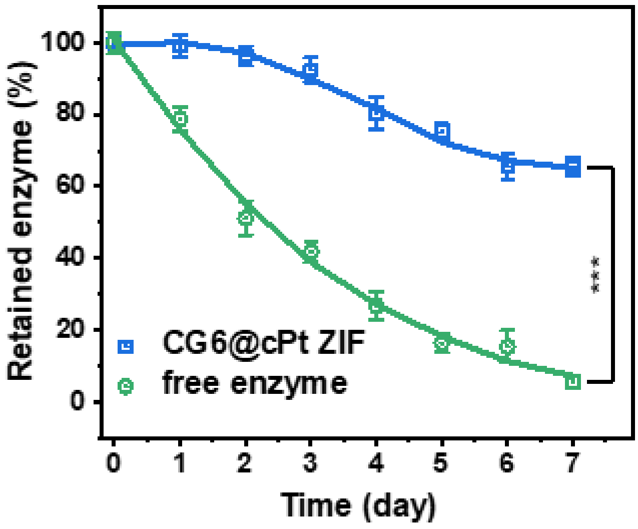

Figure S9. Retained enzyme activity of GC6@cPt ZIF nanomotors and free enzyme after stay under room temperature for several days.

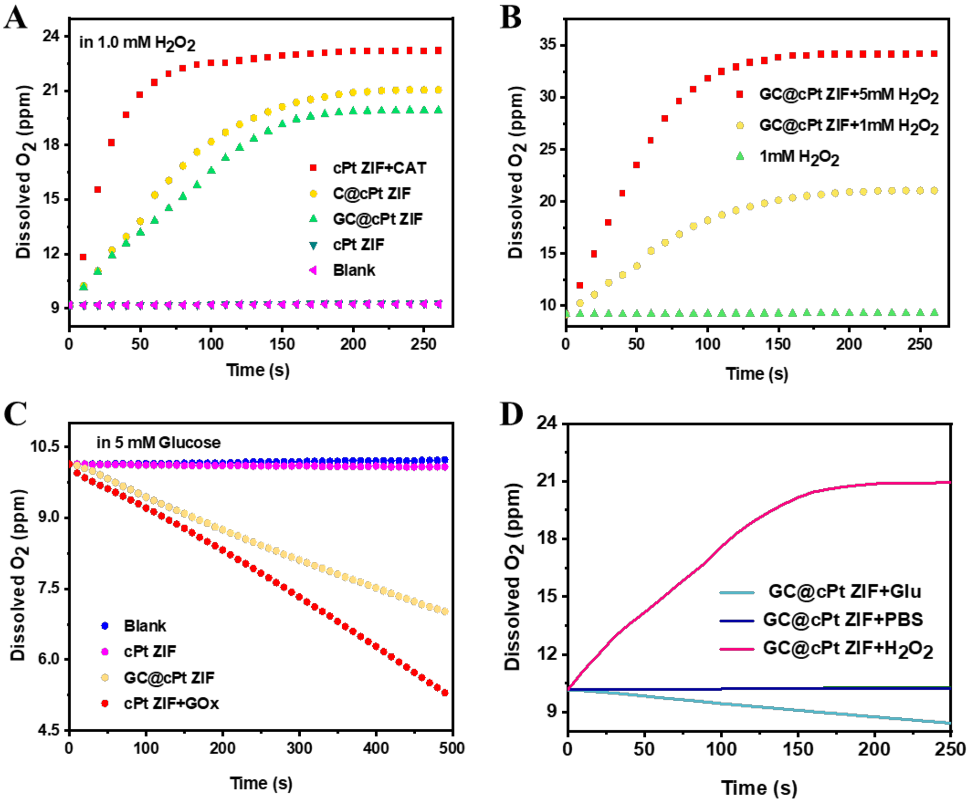

Figure S10. (A) O_2 generation 1.0 mM H_2O_2 solution after different formulations. (B) O_2 generation of GC@cPt ZIFs in H_2O_2 with different concentration. (C) The O_2 concentration changes of 5 mM glucose solution in the absence and presence of cPt ZIFs, GC@cPt ZIFs, and cPt ZIFs+GOx. (D) The O_2 concentration changes of GC@cPt ZIFs solution in the presence of PBS, glucose or H_2O_2.

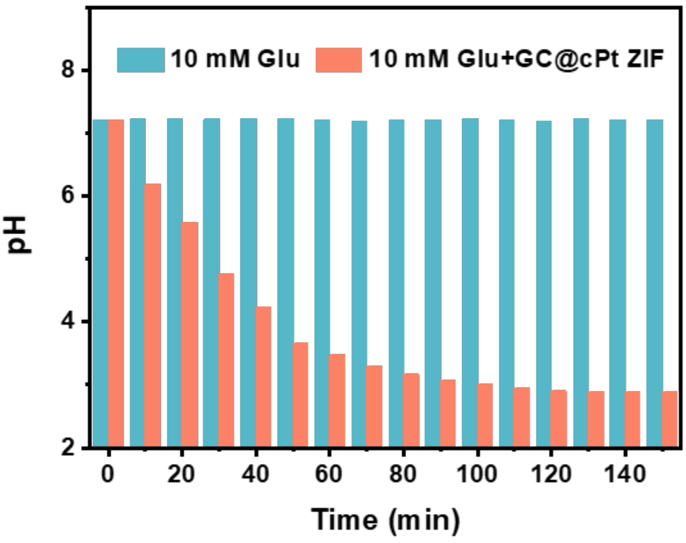

Figure S11. The pH value changes of 10 mM glucose solution in the presence and absence of GC@cPt ZIFs.

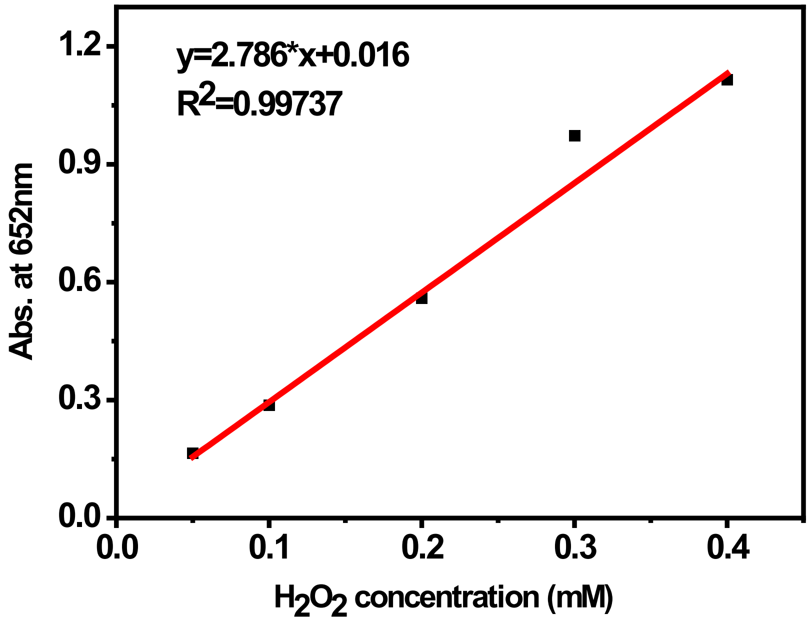

Figure S12. The standard curve of H_2O_2 concentration with HRP/TMB assay at 652 nm using UV-vis spectra .

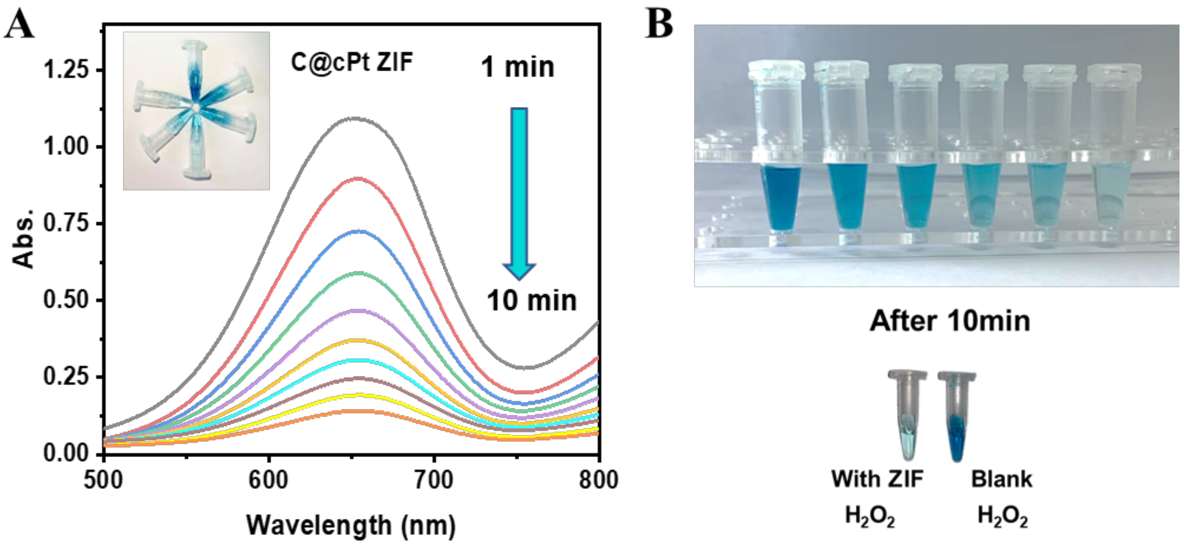

Figure S13. (A) C@cPt ZIFs incubated into the 1.0 mM H_2O_2 solution for different time. TMB/HRP assay was then added into the system for 10 min. The optical absorption spectra of TMB/HRP assay was measured versus the incubation time. (B) Photograph of the TMB/HRP colormetric assay.

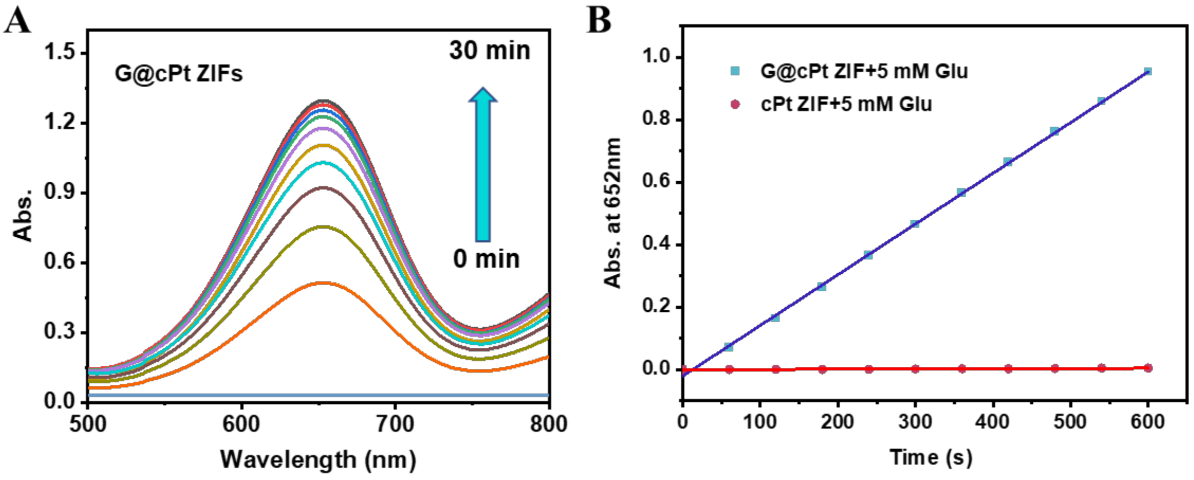

Figure S14. (A) G@cPt ZIFs incubated into the 5.0 mM glucose for different incubation time. TMB/HRP assay was then added into the system for 30 min. The optical absorption spectra of TMB/HRP assay was measured versus the incubation time. (B) Change of UV absorption at 652 nm versus incubation time for G@cPt ZIFs and cPt ZIFs.

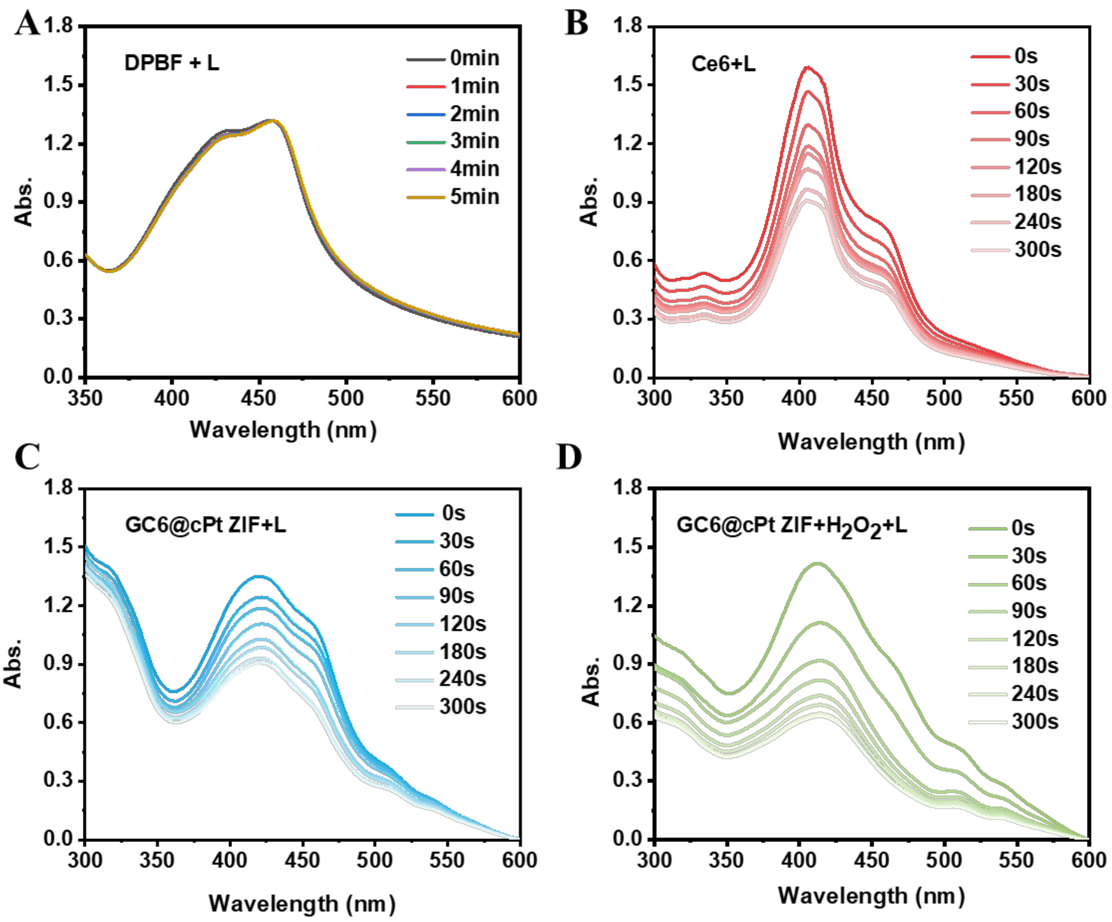

Figure S15. $^1O_2$ generation efficiency of (A) laser, (B) Ce6, (C) GC6@cPt ZIF+laser, and (D) GC6@cPt ZIF+H_2O_2+laser measured by DPBF assay at different time points. (100 µM H_2O_2 and 25 µg mL^{-1} DPBF, HAc/NaAc buffer, pH 5.0).

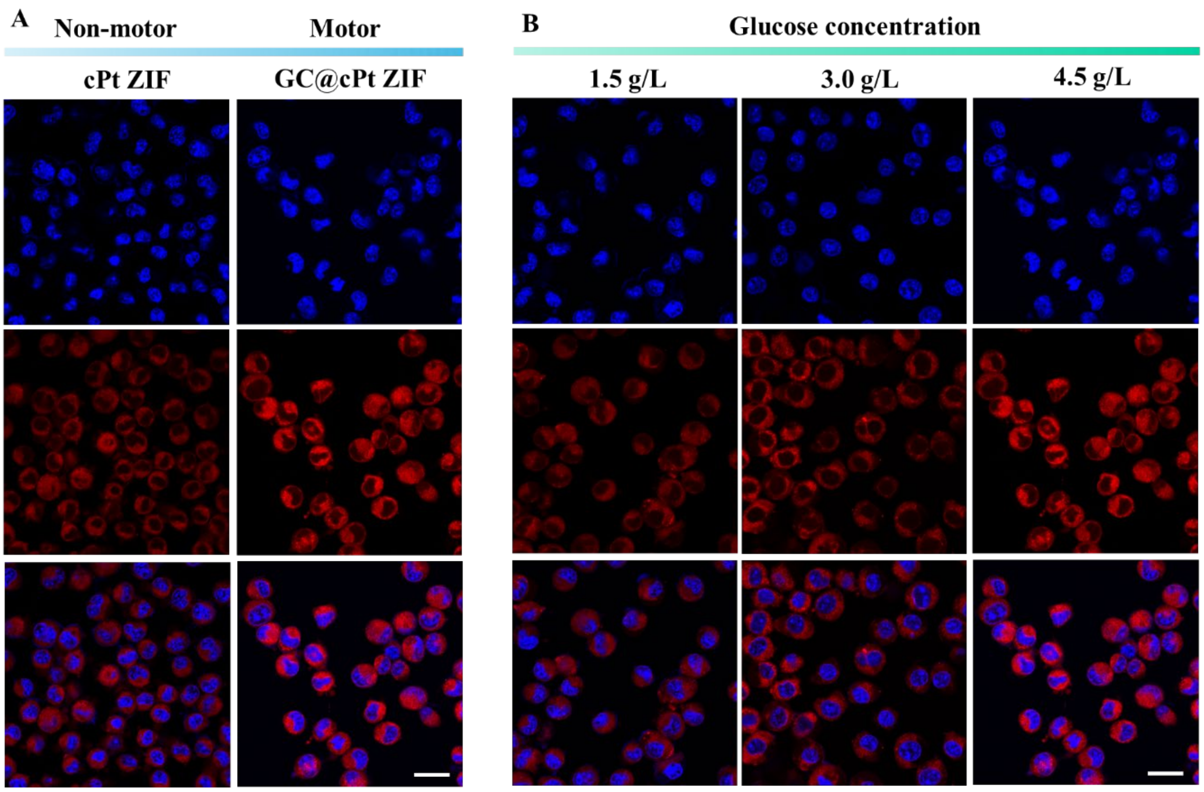

Figure S16. (A) CLSM fluorescence images of 4T1 cells when incubated with Rh B-labeled non-motor (cPt ZIF) and motor (GC@cPt ZIF). (B) CLSM fluorescence images of 4T1 cells when incubated with Rh B-labeled motor (GC@cPt ZIF) in DMEM medium with a 1.5 g/L, 3.0 g/L and 4.5 g/L of glucose concentration. The scale bar is 25 μm.

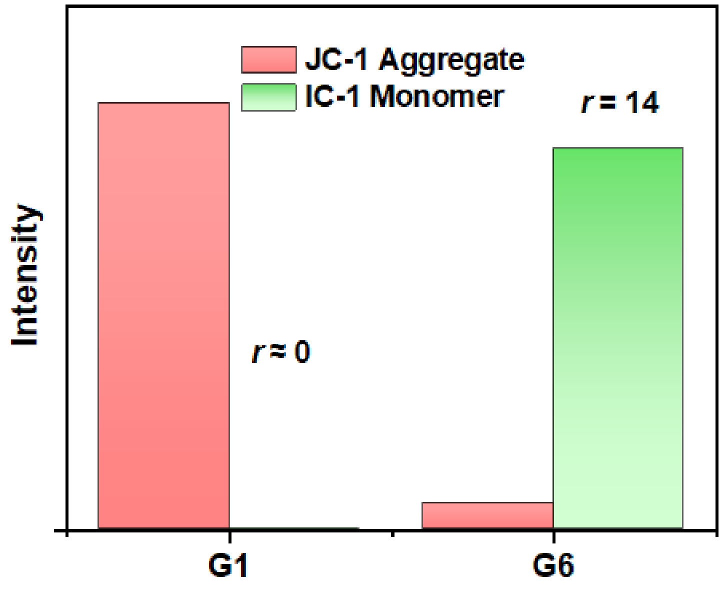

Figure S17. The ratio metric image of JC-1 monomer/aggregate value (***r***) after different treatment.

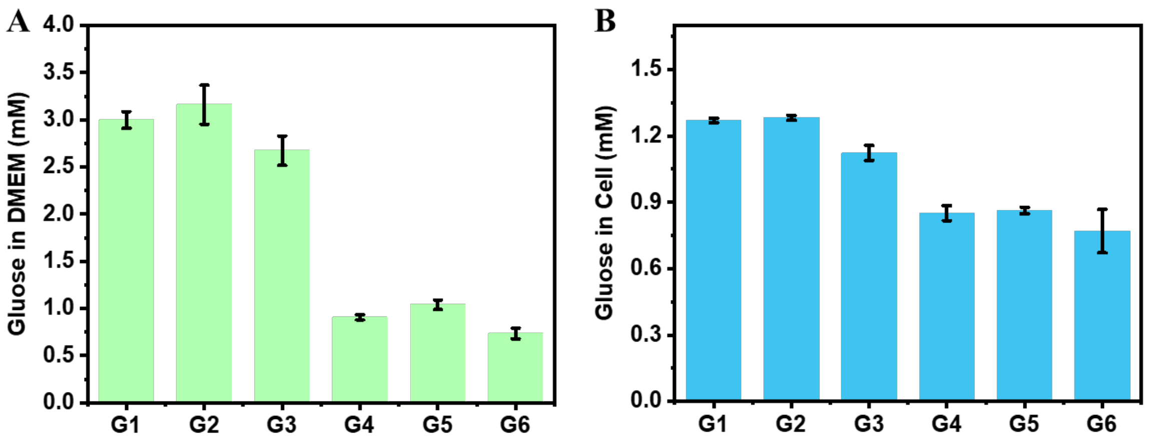

Figure S18. Glucose concentration of (A) DMEM medium and in (B) cellular when cells were incubated with PBS (G1), PBS+L (G2), cPt ZIF (G3), GC&cPt ZIF (G4), GC6@cPt motor (G5), and GC6@cPt motor+L (G6). Data represent mean ± SD (n = 5).

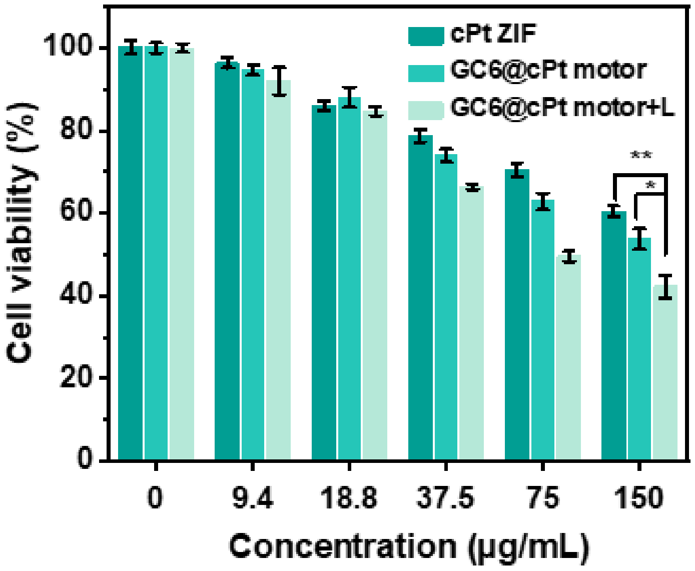

Figure S19. Relative viability of 4T1 cells cultured in low glucose DMEM (1.5 g/L) when treated with cPt ZIF, GC6@cPt ZIF motors, and GC6@cPt ZIF motors upon laser irradiation at different concentrations.

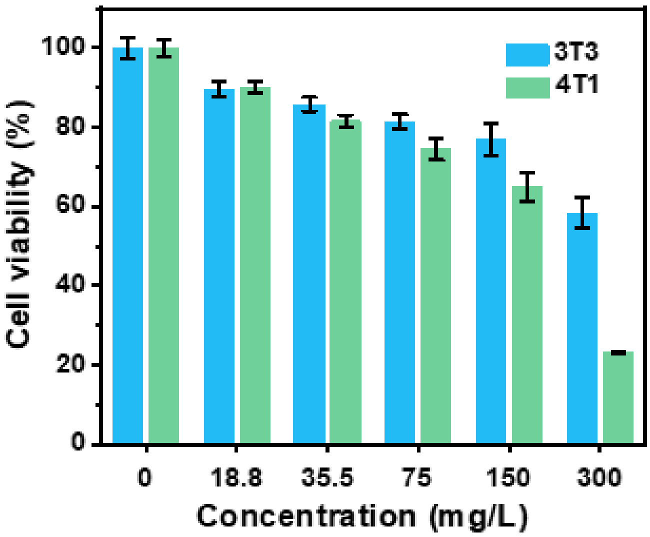

Figure S20. Relative cell viability of normal cells (3T3) and tumor cells (4T1) cultured in high glucose DMEM (4.5 g/L) when treated with cPt ZIF motors at different concentrations. Data represent mean ± SD (n = 5).

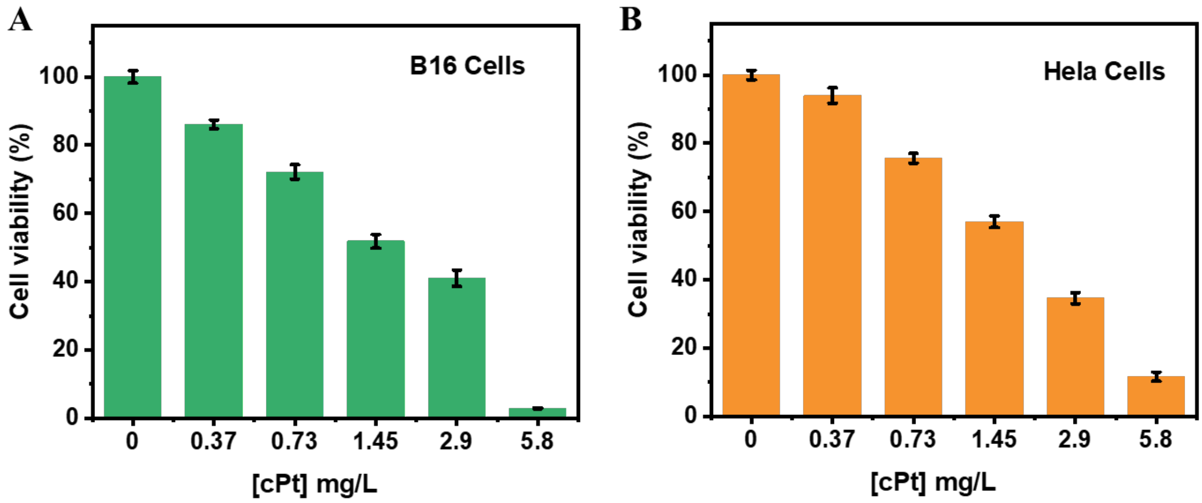

Figure S21. Relative cell viability of (A) B16 cells and (B) Hela cells cultured in high glucose DMEM (4.5 g/L) when treated with GC6@cPt ZIF motors upon laser irradiation at different concentrations. Data represent mean ± SD (n = 5).

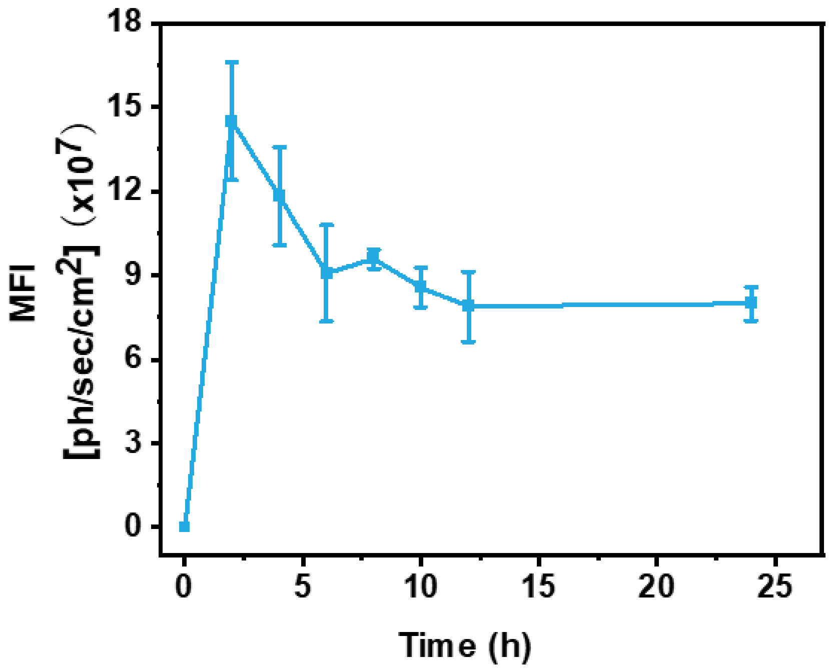

Figure S22. Quantified relative fluorescence intensity of tumor versus incubation time when 4T1-bearing mouse model was treated with GC6@cPt ZIF nanomotor group.

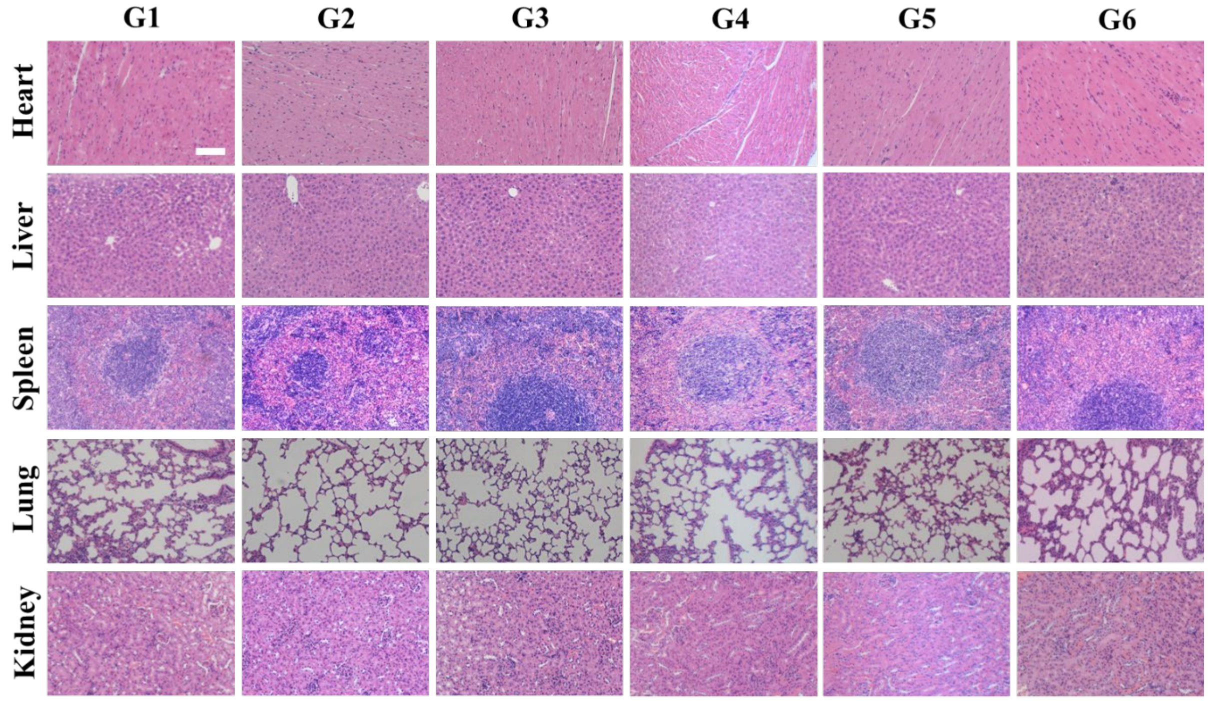

Figure S23. H&E staining images of major organs (heart, liver, spleen, lung, kidney) of the mice after injection of PBS (G1), PBS+L (G2), cPt ZIF (G3), GC&cPt ZIF (G4), GC6@cPt motor (G5), and GC6@cPt motor+L (G6). Scale bar is 100 µm.

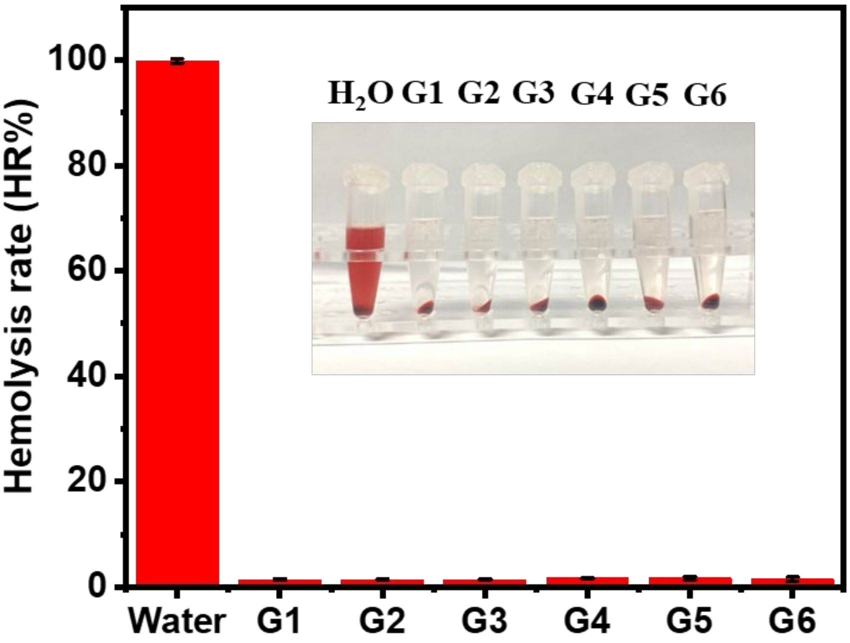

Figure S24. The hemolysis rate and the corresponding photographs with water, PBS (G1), PBS+L (G2), cPt ZIF (G3), GC&cPt ZIF (G4), GC6@cPt motor (G5), and GC6@cPt motor+L (G6).

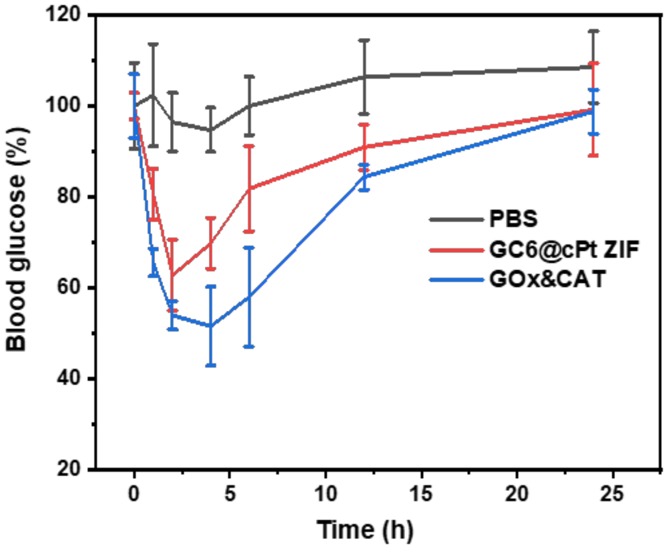

Figure S25. The blood glucose level after intravenous administration with PBS (black), GC6@cPt ZIF nanomotors (red) and binary GOx&CAT (blue).

Reference

[1] Dai, Y.; Yang, Z.; Cheng, S.; Wang, Z.; Zhang, R.; Zhu, G.; Wang, Z.; Yung, B. C.; Tian, R.; Jacobson, O.; Xu, C.; Ni, Q.; Song, J.; Sun, X.; Niu, G.; Chen, X., Toxic Reactive Oxygen Species Enhanced Synergistic Combination Therapy by Self-Assembled Metal-Phenolic Network Nanoparticles. Advanced Materials 2018, 30 (8), 1704877.

[2] Wei, D.; Yu, Y.; Zhang, X.; Wang, Y.; Chen, H.; Zhao, Y.; Wang, F.; Rong, G.; Wang, W.; Kang, X.; Cai, J.; Wang, Z.; Yin, J.-Y.; Hanif, M.; Sun, Y.; Zha, G.; Li, L.; Nie, G.; Xiao, H., Breaking the Intracellular Redox Balance with Diselenium Nanoparticles for Maximizing Chemotherapy Efficacy on Patient-Derived Xenograft Models. ACS Nano 2020, 14 (12), 16984-16996.

[3] Meng, X.; Deng, J.; Liu, F.; Guo, T.; Liu, M.; Dai, P.; Fan, A.; Wang, Z.; Zhao, Y., Triggered All-Active Metal Organic Framework: Ferroptosis Machinery Contributes to the Apoptotic Photodynamic Antitumor Therapy. Nano Letters 2019, 19 (11), 7866-7876.

[4] Tian, R.; Ma, H.; Ye, W.; Li, Y.; Wang, S.; Zhang, Z.; Liu, S.; Zang, M.; Hou, J.; Xu, J.; Luo, Q.; Sun, H.; Bai, F.; Yang, Y.; Liu, J., Se-Containing MOF Coated Dual-Fe-Atom Nanozymes With Multi-Enzyme Cascade Activities Protect Against Cerebral Ischemic Reperfusion Injury. Advanced Functional Materials n/a (n/a), 2204025.
